# Supplementary material for: Single-cell and spatial transcriptome analyses reveal tertiary lymphoid structures linked to tumour progression and immunotherapy response in nasopharyngeal carcinoma
Source: Nat Commun. 2024 Sep 4;15:7713. doi: 10.1038/s41467-024-52153-4 (PMC11375053; doi:10.1038/s41467-024-52153-4)
Supplement: Supplementary file 1 — Supplementary Information [file 41467_2024_52153_MOESM1_ESM.pdf]

sFigure 1

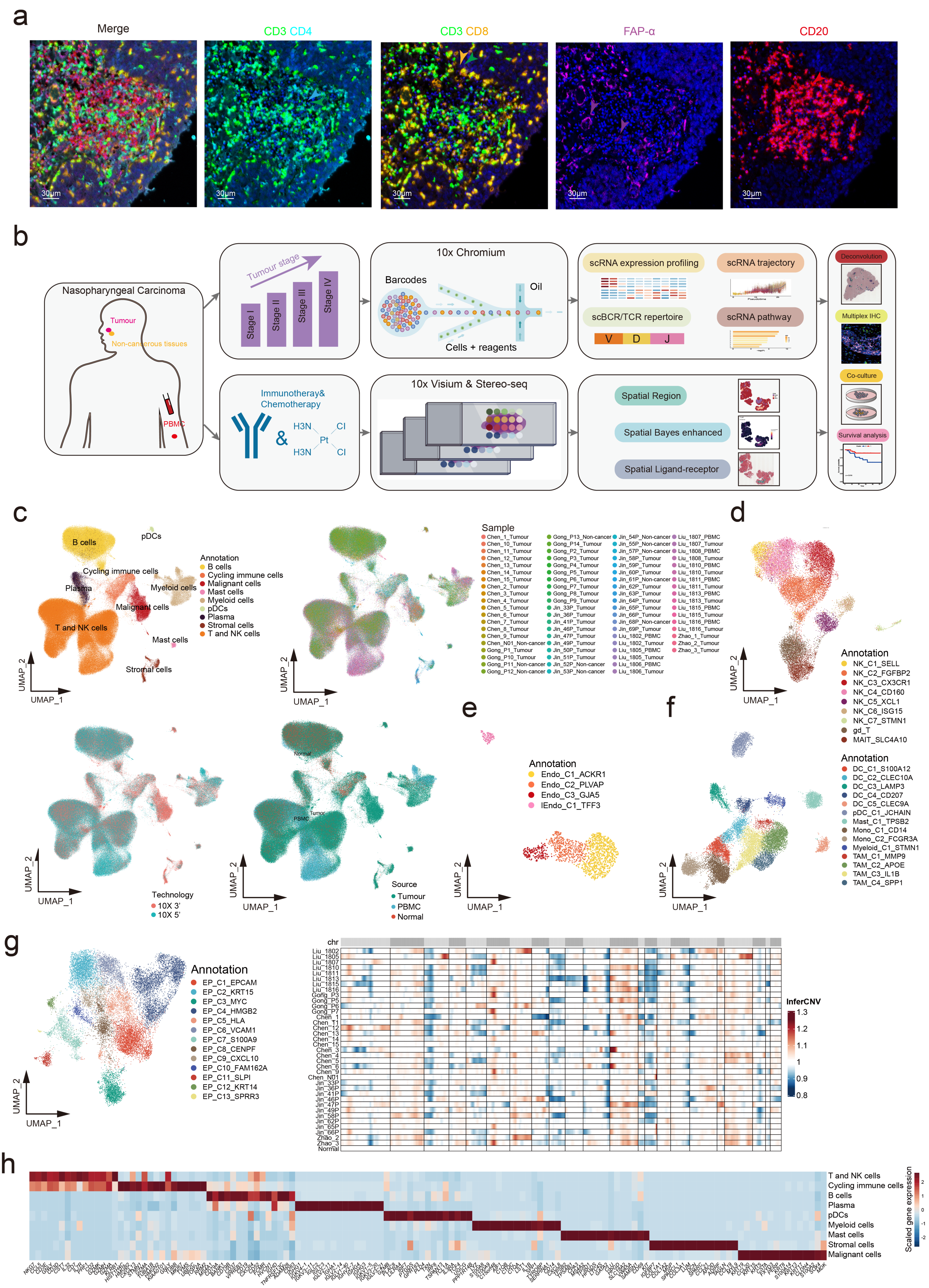
a
Merge
CD3 CD4
CD3 CD8
FAP-α
CD20
30μm
b
Nasopharyngeal Carcinoma
Tumour
Non-cancerous tissues
PBMC
Tumour stage
Stage I
Stage II
Stage III
Stage IV
Immunotheray&
Chemotherapy
10x Chromium
Barcodes
Oil
Cells + reagents
10x Visium & Stereo-seq
scRNA expression profiling
scRNA trajectory
scBCR/TCR repertoire
scRNA pathway
V
D
J
Spatial Region
Spatial Bayes enhanced
Spatial Ligand-receptor
Deconvolution
Multiplex IHC
Co-culture
Survival analysis
c
B cells
pDCs
Cycling immune cells
Plasma
Myeloid cells
Malignant cells
T and NK cells
Mast cells
Stromal cells
Annotation
Sample
Technology
10X 3'
10X 5'
Source
Tumour
PBMC
Normal
UMAP_1
UMAP_2
d
Annotation
NK_C1_SELL
NK_C2_FGFBP2
NK_C3_CX3CR1
NK_C4_CD160
NK_C5_XCL1
NK_C6_ISG15
NK_C7_STMN1
gd_T
MAIT_SLC4A10
e
Annotation
Endo_C1_ACKR1
Endo_C2_PLVAP
Endo_C3_GJA5
lEndo_C1_TFF3
f
Annotation
DC_C1_S100A12
DC_C2_CLEC10A
DC_C3_LAMP3
DC_C4_CD207
DC_C5_CLEC9A
pDC_C1_JCHAIN
Mast_C1_TPSB2
Mono_C1_CD14
Mono_C2_FCGR3A
Myeloid_C1_STMN1
TAM_C1_MMP9
TAM_C2_APOE
TAM_C3_IL1B
TAM_C4_SPP1
g
Annotation
EP_C1_EPCAM
EP_C2_KRT15
EP_C3_MYC
EP_C4_HMGB2
EP_C5_HLA
EP_C6_VCAM1
EP_C7_S100A9
EP_C8_CENPF
EP_C9_CXCL10
EP_C10_FAM162A
EP_C11_SLPI
EP_C12_KRT14
EP_C13_SPRR3
chr
InferCNV
h
T and NK cells
Cycling immune cells
B cells
Plasma
pDCs
Myeloid cells
Mast cells
Stromal cells
Malignant cells
Scaled gene expression

Supplementary Fig. 1 Basic information of the single-cell RNA sequencing

a Multiplex IHC staining of TLS in NPC tissue biopsy. Cells were coloured according to their staining with CD20 (red), CD3 (green), CD4 (cyan), CD8 (orange), and FAP-α (purple) proteins as indicated on top. The red, green, cyan, orange, and purple arrows indicated positive cells with the expression of CD20, CD3, CD4, CD8, and FAP-α proteins in NPC tissue, respectively. Images are representative of three independent samples. Scale bar is 30μm as indicated.

b Schematic diagram highlights the overall study design. First, we collected 77 scRNA-seq data for 77 samples from 56 tumour tissues and 10 peripheral blood from NPC patients, and 11 nasopharyngeal non-cancerous tissues. We also performed spatial transcriptome analyses for NPC samples using Stereo-seq (n=3) and Visium (n = 12) technologies. Next, we performed integration analyses of single-cell and spatial transcriptome data across different platforms, with clinical information available. Furthermore, we validated vital findings by multiplex IHC staining and in vitro co-culture assays. Finally, survival analyses were conducted to evaluate the clinical implication of the essential TLS components. The diagram of spatial transcriptomic model was created with BioRender.com released under a Creative Commons Attribution-NonCommercial-NoDerivs 4.0 International license.

c UMAP plots showing all the single cells identified in this study (n = 343,829). Each dot represents one cell, coloured according to major cell clusters (left top) or patients (right top) or library preparation technology (left bottom) or anatomical location (right bottom).

d, e, f UMAP plots showing NK (**d**; n = 20,051), endothelial (**e**; n = 1,550), and myeloid (**f**; n = 21,088) cell clusters identified in this study. Each dot represents one cell, coloured according to major cell clusters.

g UMAP plots of cell clusters for NPC malignant cells (n = 15,666). Each dot represents a cell, coloured according to major cell clusters (left panel). Heatmap showing the large-scale CNVs for epithelial cells (rows along y-axis) from NPC tumours. CNVs were inferred according to the average expression of 100 genes spanning each chromosomal position (x-axis). Red: gains; blue: losses. Malignant NPC cells from different patients (rows) and the range of different chromosomes are indicated as different colour bars on the top to the heatmap.

h Heatmap showing the normalized mean expression of signature genes (columns) for each cell cluster (rows). Colour from blue to red represent scaled expression levels from low to high.

sFigure 2

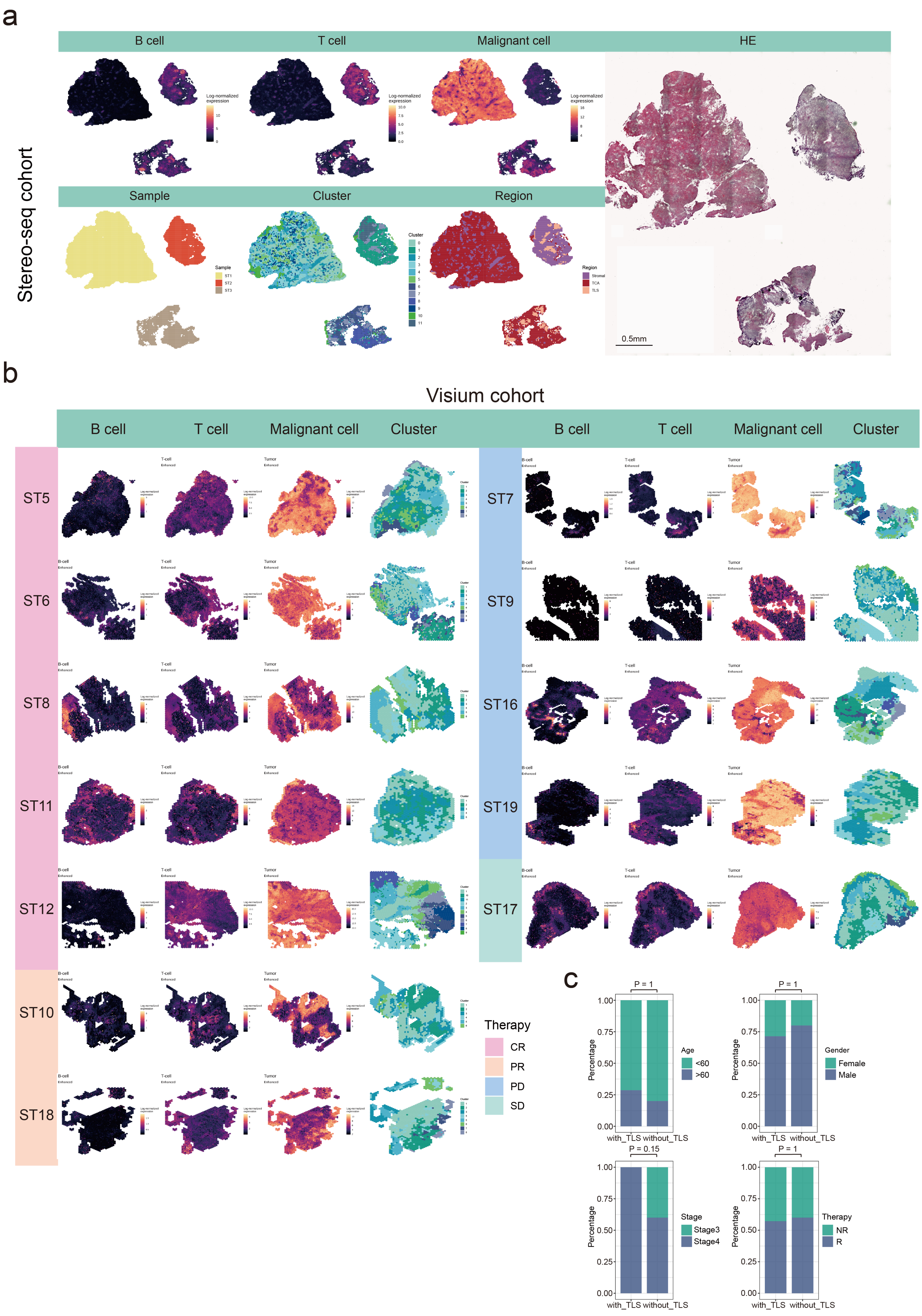
a
Stereo-seq cohort
B cell
T cell
Malignant cell
HE
Sample
Cluster
Region
Log-normalized expression
Sample
ST1
ST2
ST3
Region
Stromal
TCA
TLS
0.5mm
b
Visium cohort
B cell
T cell
Malignant cell
Cluster
ST5
ST6
ST8
ST11
ST12
ST10
ST18
ST7
ST9
ST16
ST19
ST17
Therapy
CR
PR
PD
SD
c
P = 1
P = 1
P = 0.15
P = 1
Percentage
Age
<60
>60
Gender
Female
Male
Stage
Stage3
Stage4
Therapy
NR
R
with_TLS
without_TLS

Supplementary Fig. 2 Basic information of stereo-seq and Visium cohorts

a Feature plots showing the presence and locations of B cell, T cell, malignant cell, sample, cluster, region, and HE staining in three NPC tumour biopsies for spatial transcriptome analysis using Stereo-seq (Stereo-seq cohort). The expression levels of each cell type indicated on top in each spot are filled with colours from black to yellow, representing scaled expression levels from low to high. Sample IDs are shown at ST1, ST2, and ST3. A total of 12 clusters are indicated with different colours. TLS, TCA, and Stromal regions are defined.

b Feature plots showing the presence and locations of B cell, T cell, malignant cell, and cluster in 12 NPC tumour biopsies for spatial transcriptome analysis using 10x Visium (Visium cohort). The expression levels of each cell type indicated on top in each spot are filled with colours from black to yellow, representing scaled expression levels from low to high. Sample IDs are shown at ST5, ST6, ST7, ST8, ST9, ST10, ST11, ST12, ST16, ST17, ST18, and ST19. Clusters for each sample are indicated with different colours on the cluster column. Immune response status is coloured as CR, PR, PD and SD. CR, Complete response; PD, Progressive disease; PR, Partial response; SD, Stable disease.

c Bar plots showing the proportions of NPC patients (n = 12) with distinct age, gender, stage, and immunotherapy response categories in the with TLS or without TLS group for the ST cohort. Chip-square test was performed to evaluate the significant difference. R, response; NR, non-response.

Source data are provided as a Source Data file.

sFigure 3

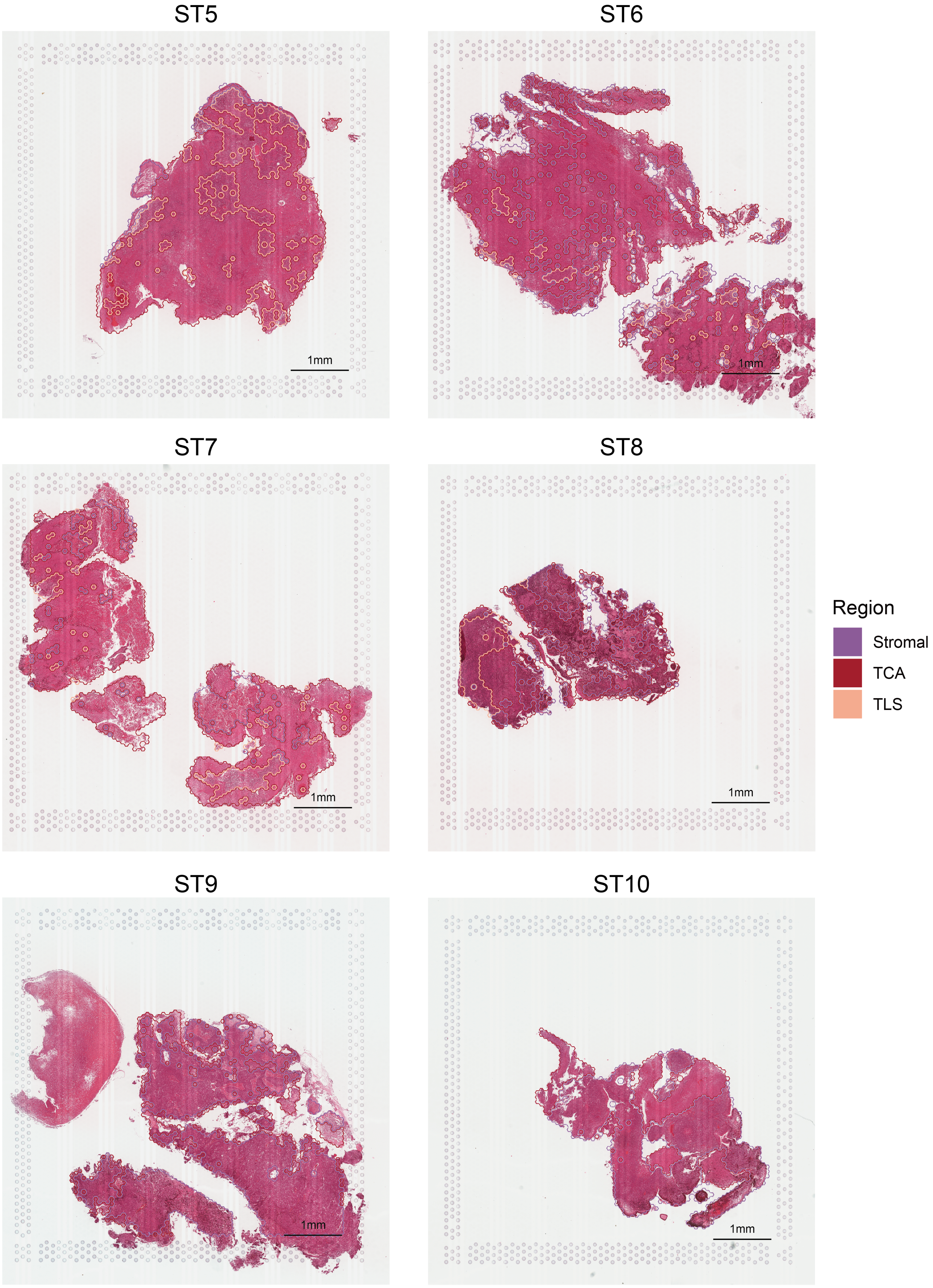
ST5
1mm
ST6
1mm
ST7
1mm
ST8
1mm
ST9
1mm
ST10
1mm
Region
Stromal
TCA
TLS

Supplementary Fig. 3 Histologic images of NPC samples for spatial transcriptomics assay.

Morphological regions were annotated by pathologists into three distinct categories: stromal tissue (purple), TLS (pink), and TCA (red). Scale bars are 1mm.

sFigure 4

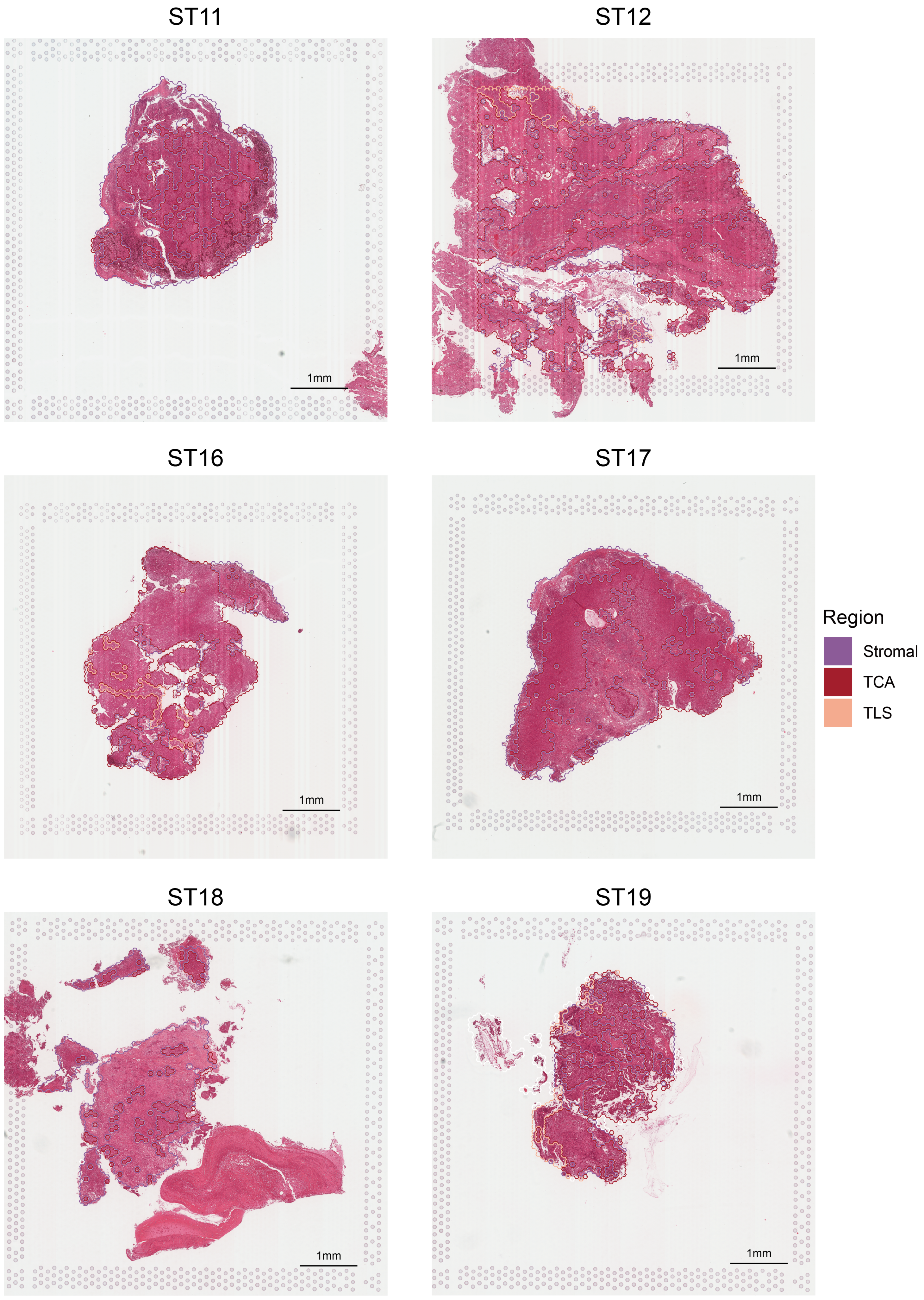
ST11
1mm
ST12
1mm
ST16
1mm
ST17
1mm
ST18
1mm
ST19
1mm
Region
Stromal
TCA
TLS

Supplementary Fig. 4 Histologic images of NPC samples for spatial transcriptomics assay.

Morphological regions were annotated by pathologists into three distinct categories: stromal tissue (purple), TLS (pink), and TCA (red). Scale bars are 1mm.

sFigure 5

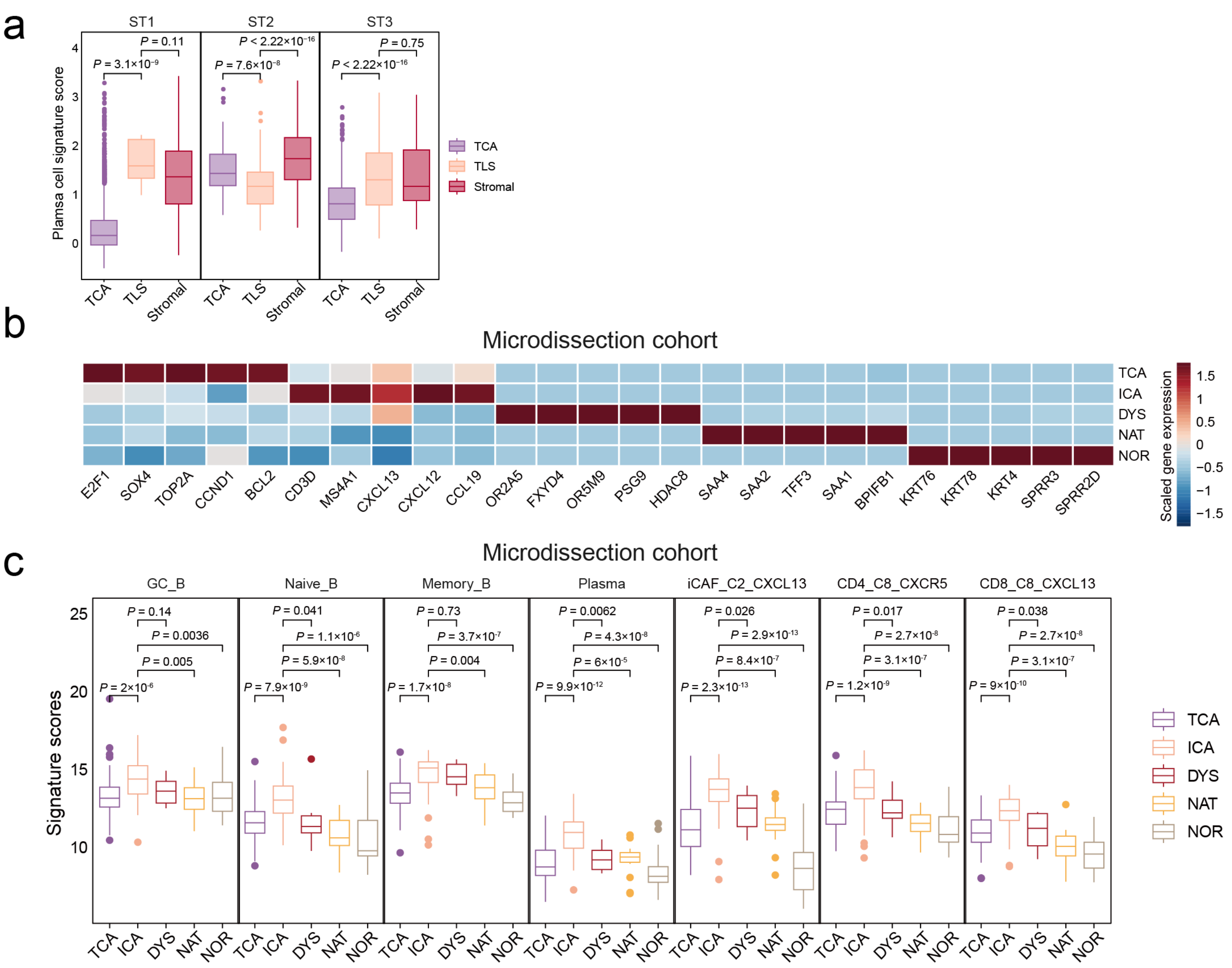
a
ST1
ST2
ST3
Plamsa cell signature score
P = 3.1×10⁻⁹
P = 0.11
P = 7.6×10⁻⁸
P < 2.22×10⁻¹⁶
P < 2.22×10⁻¹⁶
P = 0.75
TCA
TLS
Stromal
b
Microdissection cohort
TCA
ICA
DYS
NAT
NOR
E2F1 SOX4 TOP2A CCND1 BCL2 CD3D MS4A1 CXCL13 CXCL12 CCL19 OR2A5 FXYD4 OR5M9 PSG9 HDAC8 SAA4 SAA2 TFF3 SAA1 BPIFB1 KRT76 KRT78 KRT4 SPRR3 SPRR2D
Scaled gene expression
c
Microdissection cohort
GC_B
Naive_B
Memory_B
Plasma
iCAF_C2_CXCL13
CD4_C8_CXCR5
CD8_C8_CXCL13
Signature scores
TCA
ICA
DYS
NAT
NOR

Supplementary Fig. 5 Basic information of Microdissection NPC cohort.

a Box plots showing the the signature scores of plasma cells from different regions in the Stereo-seq cohort (n=9,563). In box plots, centre lines denote median values, and whiskers denote 1.5 × the interquartile range. *P* values are derived from two-sided student t-tests.

b Heatmap showing the normalized mean expression of signature genes (columns) in each microdissected tumour region (rows) in samples from the Microdissection cohort. Filled colours from blue to red represent scaled expression levels from low to high. Regions include tumour cell aggregates (TCA), immune cell aggregates (ICA), dysplastic epithelium (DYS), normal nasopharyngeal epithelium (NAT), and normal tumour-adjacent epithelium (NOR) regions.

c Box plots showing the signature scores of B (Naïve_B, Memory_B, GC_B, and plasma cells), CD4_C8_CX-CR5, CD8_C8_CXCL13, and iCAF_C2_CXCL13 cells in different microdissected regions from the Microdissection cohort (n = 189). *P* values are derived from two-sided student t-tests.

In box plots, endpoints depict minimum and maximum values; centre lines denote median values; whiskers denote 1.5 × the interquartile range. Source data are provided as a Source Data file.

sFigure 6

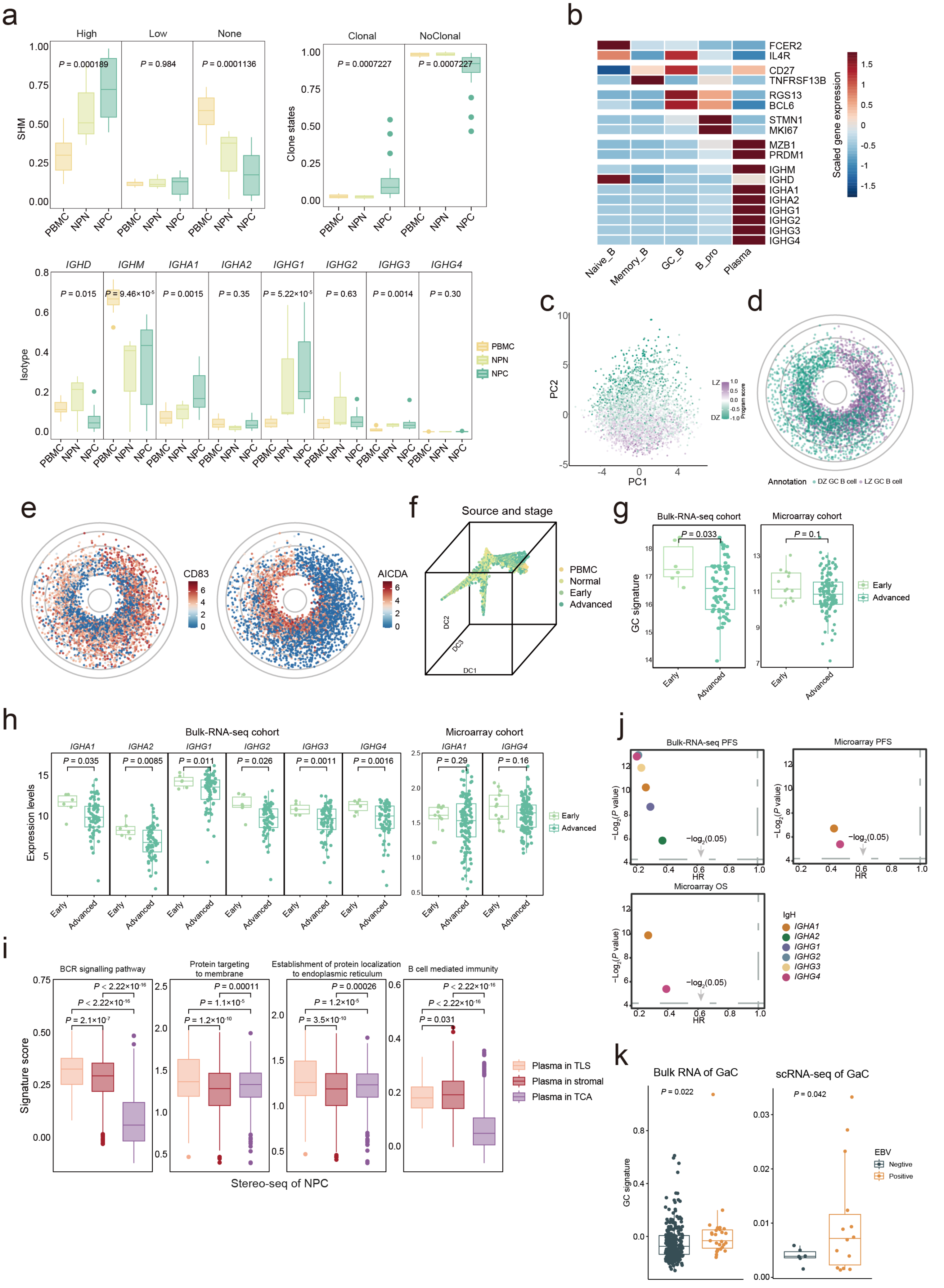

a
High
Low
None
SHM
P = 0.000189
P = 0.984
P = 0.0001136
Clonal
NoClonal
Clone states
P = 0.0007227
P = 0.0007227
PBMC
NPN
NPC
Isotype
IGHD
IGHM
IGHA1
IGHA2
IGHG1
IGHG2
IGHG3
IGHG4
b
FCER2
IL4R
CD27
TNFRSF13B
RGS13
BCL6
STMN1
MKI67
MZB1
PRDM1
Scaled gene expression
Naive_B
Memory_B
GC_B
B_pro
Plasma
c
PC1
PC2
d
Annotation
DZ GC B cell
LZ GC B cell
e
CD83
AICDA
f
Source and stage
PBMC
Normal
Early
Advanced
g
Bulk-RNA-seq cohort
Microarray cohort
GC signature
P = 0.033
P = 0.1
h
Expression levels
i
BCR signalling pathway
Protein targeting to membrane
Establishment of protein localization to endoplasmic reticulum
B cell mediated immunity
Signature score
Plasma in TLS
Plasma in stromal
Plasma in TCA
Stereo-seq of NPC
j
Bulk-RNA-seq PFS
Microarray PFS
Microarray OS
HR
IgH
k
Bulk RNA of GaC
scRNA-seq of GaC
P = 0.022
P = 0.042
EBV
Negtive
Positive

Supplementary Fig. 6 Clinical implications of B cell and antibody production in NPC.

a Box plots showing the SHM, clone states (top panel), and isotype of B cells among PBMC, NPN, and NPC (bottom panel; n = 34). The comparison was made using two-sided Kruskal-Wallis test across PBMC, NPN, and NPC.

b Heatmap showing the normalized mean expression of B cell-related genes (rows) in different B cell clusters (columns). Filled colours from blue to red represent scaled expression levels from low to high.

c Radial projection of PC1×PC2 cartesian coordinates highlights a circular trajectory colouring according light or dark zone score.

d,e Circular plot showing cell clusters (**d**) and the expression levels of light zone (CD83) and dark zone (AICDA; **e**). Filled colours from blue to red represent scaled expression levels from low to high.

f 3D plot showing the spatial distribution of B cell clusters. Each dot represents one single cell, coloured according to its sample source (PBMC or Normal tissue) and clinical stage (early or advanced) as indicated.

g Box plots showing the GC signature in samples from the Bulk-RNA-seq (n = 59) and Microarray (n = 159) cohorts. Samples were grouped into early and advanced stages.

h Box plots showing the expression levels of IgH (*IGHD*, *IGHM*, *IGHA1*, *IGHA2*, *IGHG1*, *IGHG2*, *IGHG3*, and *IGHG4*) in samples from the Bulk-RNA-seq (n = 59) and Microarray cohorts (n = 159). Samples were grouped into early and advanced stages.

i Box plots showing the expression levels of antibody production and immune response-related pathways of plasma cells from different regions in the Stereo-seq cohort.

j Survival analysis of IgH (IGHD, IGHM, IGHA1, IGHA2, IGHG1, IGHG2, IGHG3, and IGHG4) transcriptome in two NPC cohorts (Bulk-RNA-seq, n = 147; and Microarray, n = 150). *P* value (y-axis) and HR (x-axis) were calculated using two-sided cox tests and plotted in different colours for each gene annotated at the right. PFS, progression-free survival; OS, overall survival.

k Box plots showing the GC B cell signature (left panel) and proportion (right panel) in samples from the Bulk-RNA-seq (left panel; TCGA data; n = 277) and scRNA-seq (right panel; n = 20) GaC cohorts, respectively. The GC B cell signature and groups are indicated at the y- and x-axis, respectively.

In box plots, endpoints depict minimum and maximum values; centre lines denote median values; whiskers denote 1.5 × the interquartile range. The comparison was made using two-sided t-test. Source data are provided as a Source Data file.

sFigure 7

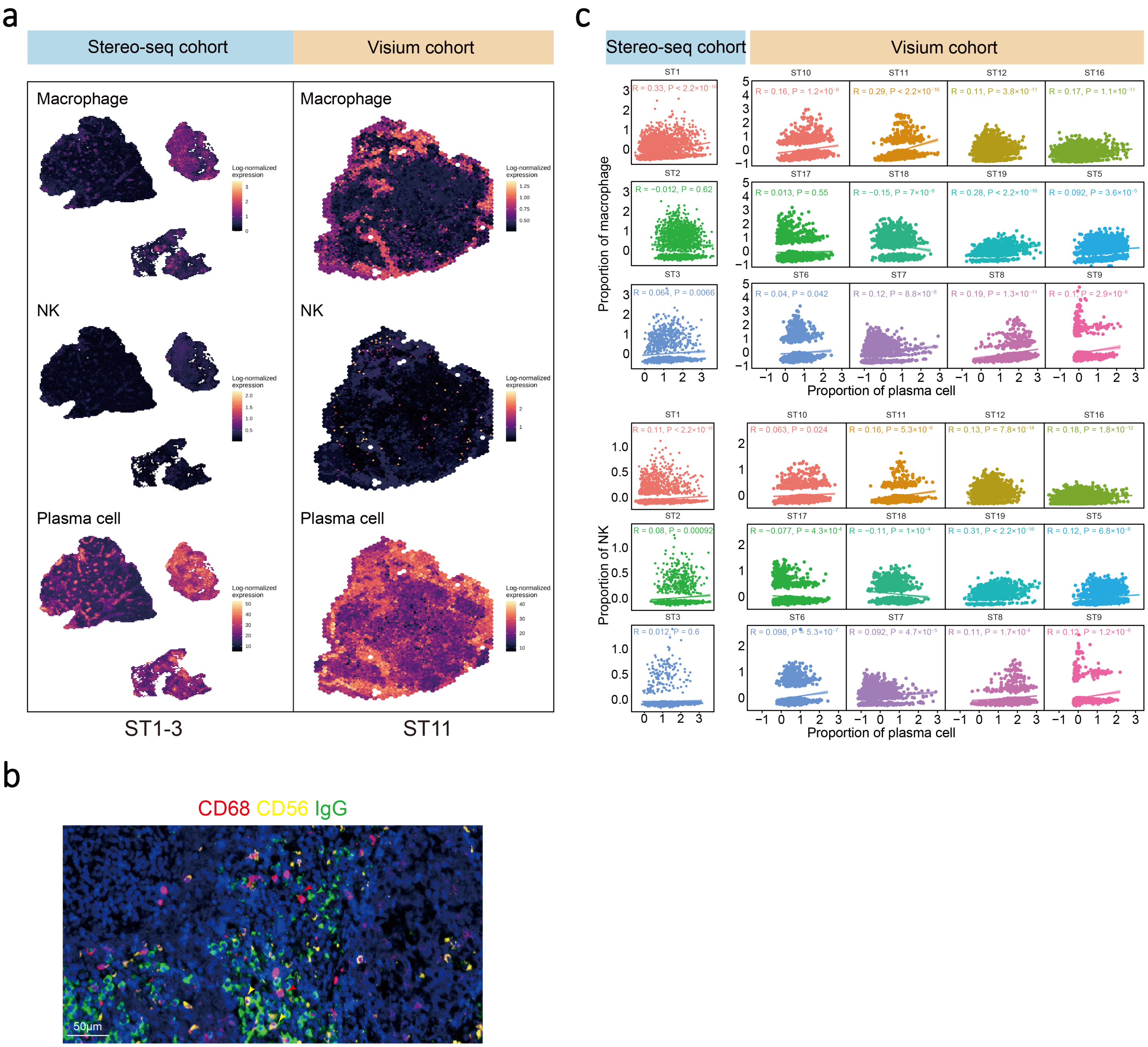
a
Stereo-seq cohort
Visium cohort
Macrophage
NK
Plasma cell
Log-normalized expression
ST1-3
ST11
b
CD68 CD56 IgG
50μm
c
Stereo-seq cohort
Visium cohort
Proportion of macrophage
Proportion of NK
Proportion of plasma cell
ST1
ST2
ST3
ST10
ST11
ST12
ST16
ST17
ST18
ST19
ST5
ST6
ST7
ST8
ST9

Supplementary Fig. 7 ADCC and ADCP in NPC.

a Representative images of spatial feature plots showing the expression levels of macrophage, NK cells, and pan-Ig signatures in each spot of tumour samples from the Stereo-seq and the Visium cohorts. Filled colours from black to yellow represent scaled expression levels from low to high.

b Representative image of multiplex IHC staining of NPC tissue biopsy. Cells were coloured according to their staining with IgG (green), CD56 (yellow), and CD68 (red) proteins as indicated on top. The green, yellow, and red arrows indicated positive cells with the expression of IgG, CD56, and CD68 proteins in NPC tissue, respectively. Images are representative of three independent samples. Scale bar is 50μm as indicated.

c Scatter plots showing the correlations of the proportions between plasma cells and macrophage (top) or NK cells (bottom) in NPC samples from the Stereo-seq or the Visium cohorts. The correlation was assessed using Pearson’s correlation test.

sFigure 8

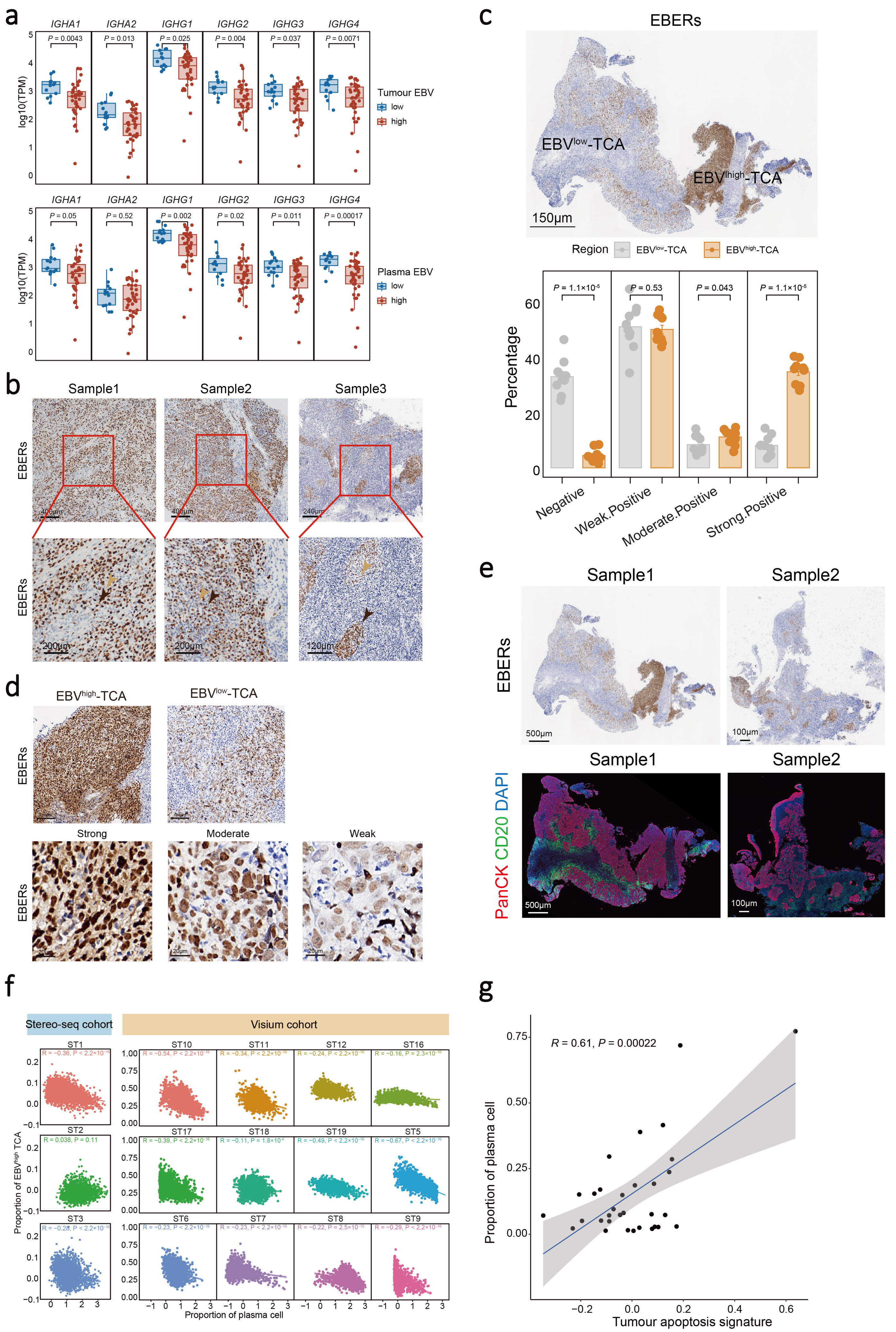
a
IGHA1
IGHA2
IGHG1
IGHG2
IGHG3
IGHG4
P = 0.0043
P = 0.013
P = 0.025
P = 0.004
P = 0.037
P = 0.0071
log10(TPM)
Tumour EBV
low
high
P = 0.05
P = 0.52
P = 0.002
P = 0.02
P = 0.011
P = 0.00017
Plasma EBV
low
high
b
Sample1
Sample2
Sample3
EBERs
400μm
400μm
240μm
200μm
200μm
120μm
c
EBERs
EBVlow-TCA
EBVhigh-TCA
150μm
Region
EBVlow-TCA
EBVhigh-TCA
P = 1.1×10-5
P = 0.53
P = 0.043
P = 1.1×10-5
Percentage
Negative
Weak.Positive
Moderate.Positive
Strong.Positive
d
EBVhigh-TCA
EBVlow-TCA
Strong
Moderate
Weak
e
Sample1
Sample2
500μm
100μm
PanCK CD20 DAPI
f
Stereo-seq cohort
Visium cohort
ST1
ST10
ST11
ST12
ST16
ST2
ST17
ST18
ST19
ST5
ST3
ST6
ST7
ST8
ST9
Proportion of EBVhigh TCA
Proportion of plasma cell
g
R = 0.61, P = 0.00022
Proportion of plasma cell
Tumour apoptosis signature

Supplementary Fig. 8 EBER staining and expression level of EBV and apoptosis genes

a Box plots showing the expression levels of IgH (*IGHD*, *IGHM*, *IGHA1*, *IGHA2*, *IGHG1*, *IGHG2*, *IGHG3*, and *IGHG4*) in samples (n = 59) from the Bulk-RNA-seq cohort. Samples were grouped according to the EBV load (high/low) in tumour (top) or peripheral blood (bottom). coloured dots denote each patient. The comparison was made using two-sided t-test.

b Representative images of EBERs staining with different intensity in NPC biopsy tissue samples. The dark brown arrows represent the EBV-high state, while the light brown arrows represent the EBV-low state. Scale bars are 400μm and 240μm for the top panel and 200μm and 120μm for the bottom panel.

c Representative image of the in-situ hybridization of EBERs in NPC biopsy section (top panel). Brown staining indicates scattered EBER-positive malignant cells in NPC tissue. Images are representative of three independent samples. Scale bar is 150μm. Bar plot (bottom panel) showing the percentage of different EBERs staining cells in EBV^{low}- and EBV^{high}-TCA (n = 20). *P* values are derived from two-sided student t-tests. The data is presented as mean ± SD.

d Representative image of the in-situ hybridization of EBERs in NPC biopsy section. Brown staining indicates scattered EBER-positive malignant cells in NPC tissue. Images are representative of three independent samples. Scale bars for the top and bottom panels are 100μm and 20μm, respectively. Representative images of EBERs staining with different intensity (strong, moderate, and weak) in NPC biopsy tissue samples were showing.

e Results of in-situ hybridization (top) and multiplex IHC staining (bottom) in NPC biopsy sections. Brown staining on the top panels indicates scattered EBER-positive malignant cells in NPC tissue. Cells in the bottom panels were coloured according to their staining with PanCK (red) and CD20 (green) proteins as indicated. Images are representative of three independent samples. Scale bars are 500μm and 100μm as indicated.

f Scatter plots showing the correlation of the proportions between plasma cells and EBV^{high}-TCA in NPC samples from the Stereo-seq or Visium cohorts. The correlation was assessed using Pearson's correlation test.

g Scatter plots showing the pair-wise correlation of tumour apoptotic signature (x-axis) and the proportion of plasma cells (y-axis) in the NPC scRNA-seq cohort. 32 NPC samples were included, shown as dots. R value represents Pearson's correlation. P value is calculated by two-sided Pearson correlation analysis.

In box plots, endpoints depict minimum and maximum values; centre lines denote median values; whiskers denote 1.5 × the interquartile range. Source data are provided as a Source Data file.

sFigure 9

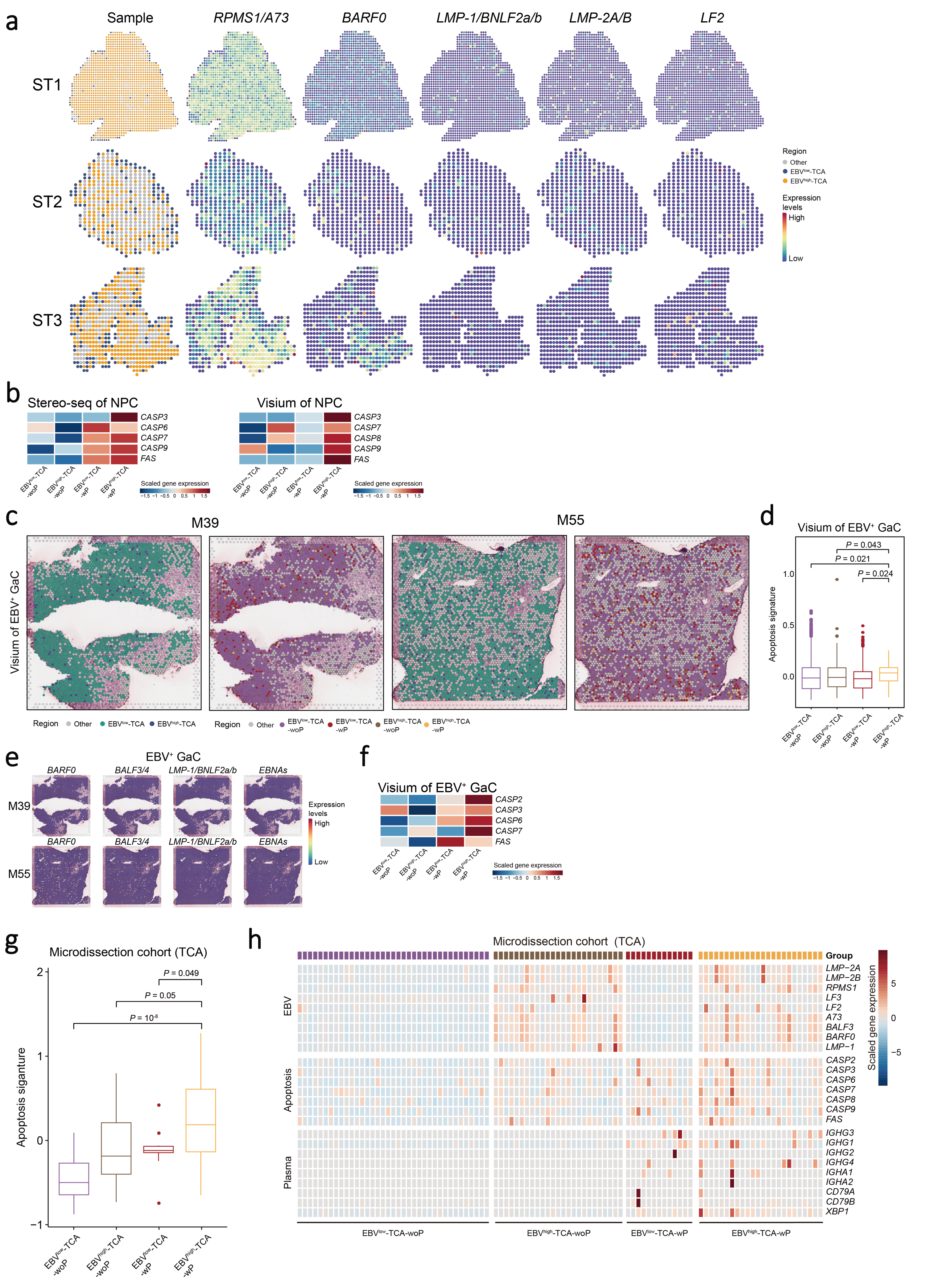
a
Sample
RPMS1/A73
BARF0
LMP-1/BNLF2a/b
LMP-2A/B
LF2
ST1
ST2
ST3
Region
Other
Expression levels
High
Low
b
Stereo-seq of NPC
Visium of NPC
CASP3
CASP6
CASP7
CASP9
FAS
CASP8
Scaled gene expression
c
M39
M55
Visium of EBV⁺ GaC
Region
Other
d
Visium of EBV⁺ GaC
P = 0.043
P = 0.021
P = 0.024
Apoptosis signature
e
EBV⁺ GaC
BARF0
BALF3/4
LMP-1/BNLF2a/b
EBNAs
f
Visium of EBV⁺ GaC
CASP2
g
Microdissection cohort (TCA)
P = 0.049
P = 0.05
Apoptosis signature
h
Microdissection cohort (TCA)
Group
EBV
Apoptosis
Plasma
LMP−2A
LMP−2B
RPMS1
LF3
LF2
A73
BALF3
BARF0
LMP−1
IGHG3
IGHG1
IGHG2
IGHG4
IGHA1
IGHA2
CD79A
CD79B
XBP1
Scaled gene expression

Supplementary Fig. 9 Additional characteristics of TCA

a Representative images of spatial feature plots showing the expression levels of EBV molecules in NPC tumours from the Stereo-seq cohorts. EBV-encoded genes are indicated on top. Filled colours from blue to red represent scaled expression levels from low to high.

b Heatmap showing the normalized mean expression of apoptosis-related genes (rows) for different TCA compartments (columns) based on EBV and plasma cell contact in NPC samples from the Stereo-seq (left) or the Visium (right) cohorts. Filled colours from blue to red represent scaled expression levels from low to high.

c Spatial distribution of different TCA in representative EBV^+ GaC sections from the GaC-Visium cohort. Each type of TCA is coloured as indicated.

d Box plots showing the apoptosis signatures in different TCA of EBV^+ GaC (n = 4,525).

e Representative images of spatial feature plots showing the expression levels of EBV molecules in EBV^+ GaC from the Stereo-seq cohorts. EBV-encoded genes are indicated on top. Filled colours from blue to red represent scaled expression levels from low to high.

f Heatmap showing the normalized mean expression of apoptosis-related genes (rows) for different TCA compartments (columns, as defined in **c**) in EBV^+ GaC samples of the GaC-Visium cohort. Filled colours from blue to red represent scaled expression levels from low to high.

g Box plots showing the apoptosis signatures in different TCA compartments in NPC samples (n = 96) from the Microdissection cohort.

h Heatmap showing the normalized mean expression of EBV, apoptosis, and plasma-related genes (rows) for different TCA compartments (columns) in the Microdissection cohort. Filled colours from blue to red represent scaled expression levels from low to high.

In box plots, endpoints depict minimum and maximum values; centre lines denote median values; whiskers denote 1.5 × the interquartile range. The comparison was made using two-sided t-test. Source data are provided as a Source Data file.

sFigure 10

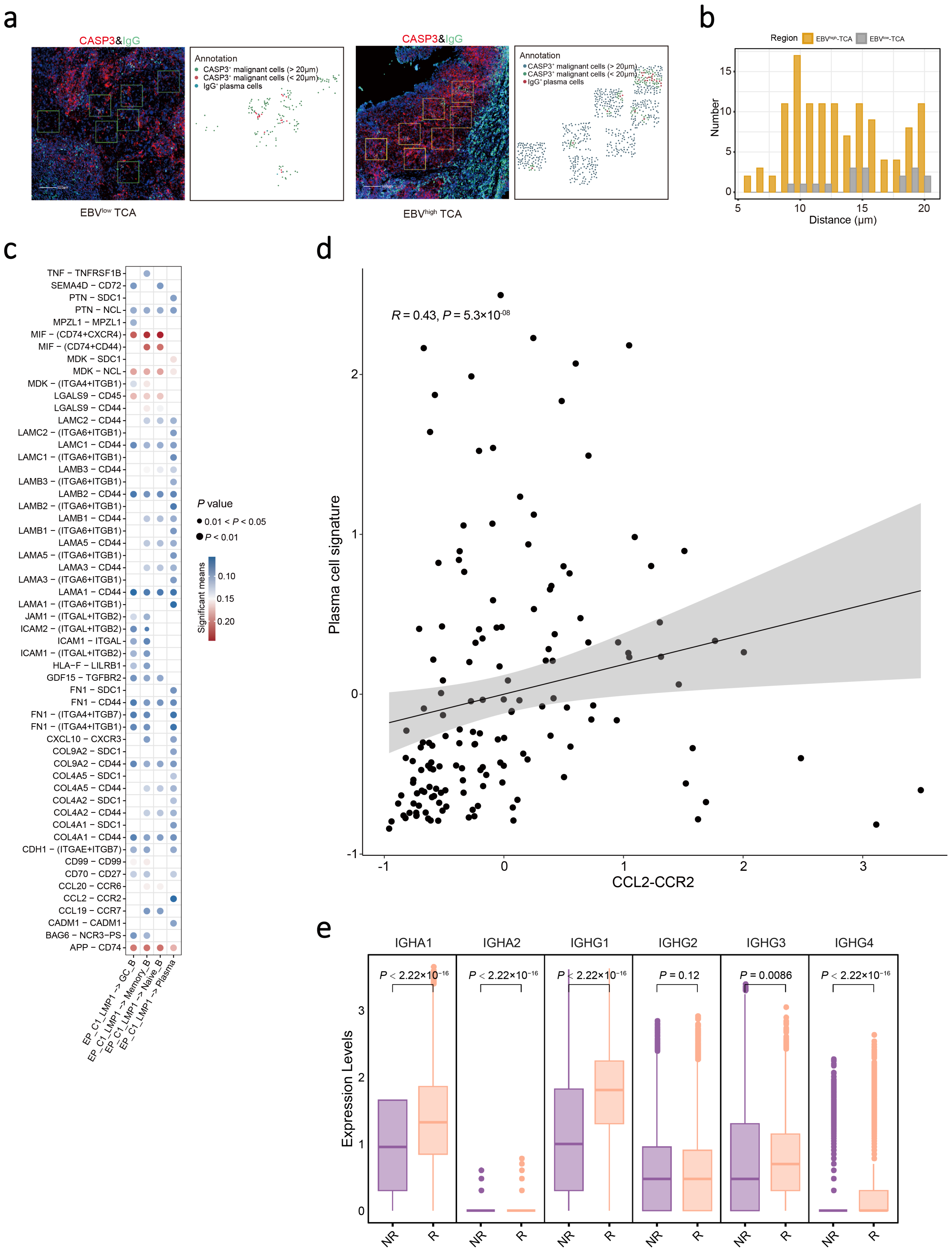

a
CASP3&IgG
Annotation
CASP3+ malignant cells (> 20μm)
CASP3+ malignant cells (< 20μm)
IgG+ plasma cells
EBVlow TCA
EBVhigh TCA
b
Region EBVhigh-TCA EBVlow-TCA
Number
Distance (μm)
c
P value
0.01 < P < 0.05
P < 0.01
Significant means
EP_C1_LMP1 -> GC_B
EP_C1_LMP1 -> Memory_B
EP_C1_LMP1 -> Naive_B
EP_C1_LMP1 -> Plasma
d
R = 0.43, P = 5.3×10-08
Plasma cell signature
CCL2-CCR2
e
IGHA1
IGHA2
IGHG1
IGHG2
IGHG3
IGHG4
P < 2.22×10-16
P = 0.12
P = 0.0086
Expression Levels
NR
R

Supplementary Fig. 10 Distance and interaction of EBV^+ NPC malignant cell and plasma cells.

a Representative image of multiplex IHC staining for CASP3 and IgG in NPC tissue biopsy (left panel). Cell markers, including CASP3 (red) and IgG (green) proteins, are coloured using respective antibodies. Images are representative of three independent samples. Scale bar is 100μm. Dot plots showing the distribution of $CASP3^+$ malignant and IgG^+ plasma cells, coloured by cell cluster and distance (right panel).

b Bar plot showing the distribution of $CASP3^+$ malignant cells in relation to their distance from IgG^+ plasma cells.

c Cell-cell interactions between EBV^+ (LMP1) malignant epithelial cells and B cells (n = 10,589) in NPC. Significant ligand-receptor pairs contributing to the signalling from EBV^+ malignant cell to four B cell clusters. The dot colour and size represent the calculated communication probability and *P* values, respectively. *P* values are derived from one-sided permutation test. Colour from blue to red indicates communication probability from low to high.

d Scatter plot showing the correlation between the probability of CCL-CCR2 mediated communication and the plasma cell signature in Bulk RNA-seq cohort (n = 147). Each data point represents an individual sample. Pearson correlation coefficient was used to assess the relationship, and the confidence interval of the correlation is depicted in grey.

e Box plots showing the expression levels of IgH (*IGHD*, *IGHM*, *IGHA1*, *IGHA2*, *IGHG1*, *IGHG2*, *IGHG3*, and *IGHG4*) in the responders (R) and non-responders (NR) to ICB (toripalimab plus chemotherapy) treatment from the Visium cohort of NPC (n=12). *P* values are derived from two- sided student t-tests.

In box plots, endpoints depict minimum and maximum values; centre lines denote median values; whiskers denote 1.5 × the interquartile range. Source data are provided as a Source Data file.

sFigure 11

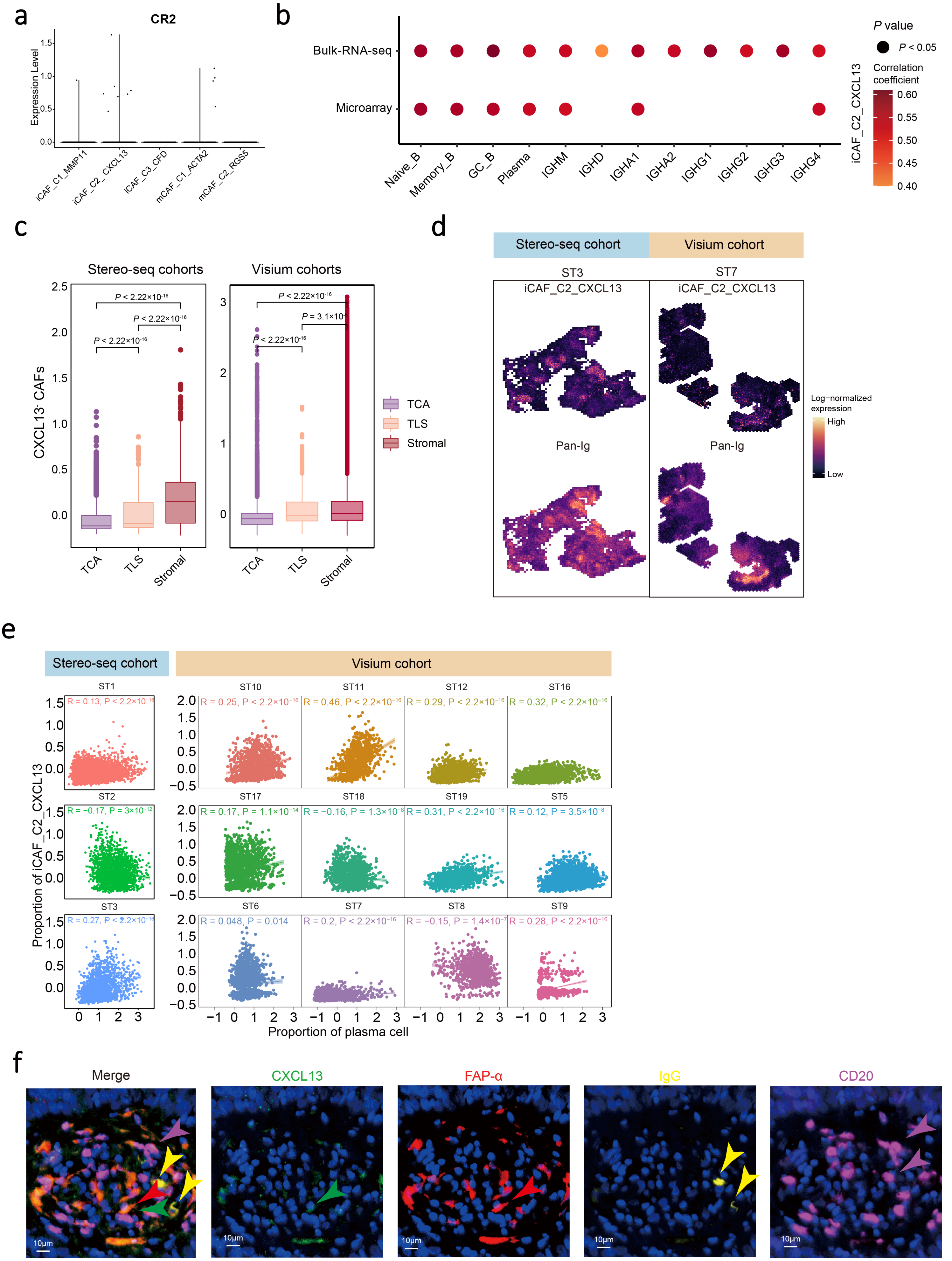
a
CR2
Expression Level
iCAF_C1_MMP11
iCAF_C2_CXCL13
iCAF_C3_CFD
mCAF_C1_ACTA2
mCAF_C2_RGS5
b
Bulk-RNA-seq
Microarray
Naive_B
Memory_B
GC_B
Plasma
IGHM
IGHD
IGHA1
IGHA2
IGHG1
IGHG2
IGHG3
IGHG4
iCAF_C2_CXCL13
P value
P < 0.05
Correlation coefficient
c
Stereo-seq cohorts
Visium cohorts
CXCL13- CAFs
TCA
TLS
Stromal
d
Stereo-seq cohort
Visium cohort
ST3
ST7
iCAF_C2_CXCL13
Pan-Ig
Log−normalized expression
High
Low
e
Stereo-seq cohort
Visium cohort
Proportion of iCAF_C2_CXCL13
Proportion of plasma cell
f
Merge
CXCL13
FAP-α
IgG
CD20
10μm

Supplementary Fig. 11 Correlation of iCAF_C2_CXCL13 CAFs and pan-Ig.

a Violin plot showing the expression levels of *CR2* in different clusters of fibroblasts (n = 1,652).

b Dot plots showing the correlation of iCAF_C2_CXCL13 signature scores with the expression levels of IgH (*IGHD*, *IGHM*, *IGHA1*, *IGHA2*, *IGHG1*, *IGHG2*, *IGHG3*, and *IGHG4*) in samples from the Bulk-RNA-seq (top) and the Microarray (bottom) cohorts. The correlation was assessed using Pearson's correlation test and indicated as heatmap colours.

c Box plots showing the signature scores of $CXCL13^-$ CAFs in different tumour regions from NPC patients of the Stereo-seq (n = 9,563; left) and the Visium cohorts (n = 21,753; right). *P* values are derived from two-sided student t-tests.

d Representative images of spatial feature plots showing the expression levels of iCAF_C2_CXCL13 and pan-Ig signatures in each spot of tumour samples from the Stereo-seq or the Visium cohorts. Filled colours from black to yellow represent scaled expression levels from low to high.

e Scatter plots showing the correlation of iCAF_C2_CXCL13 signature scores and pan-Ig signature scores in NPC samples from the Stereo-seq (n = 9,563) or the Visium (n = 21,753) cohorts. The correlation was assessed using Pearson's correlation test.

f Representative image of multiplex IHC staining of $CXCL13^+$ CAFs in NPC tissue biopsy. Cells were coloured according to their staining with CXCL13 (green), IgG (yellow), CD20 (purple), and FAP-α (red) proteins as indicated on top. The green, yellow, purple, and red arrows indicated positive cells with the expression of CXCL13, IgG, CD20, and FAP-α proteins in NPC tissue, respectively. Images are representative of three independent samples. Scale bar is 10μm as indicated.

In box plots, endpoints depict minimum and maximum values; centre lines denote median values; whiskers denote 1.5 × the interquartile range. Source data are provided as a Source Data file.

sFigure 12

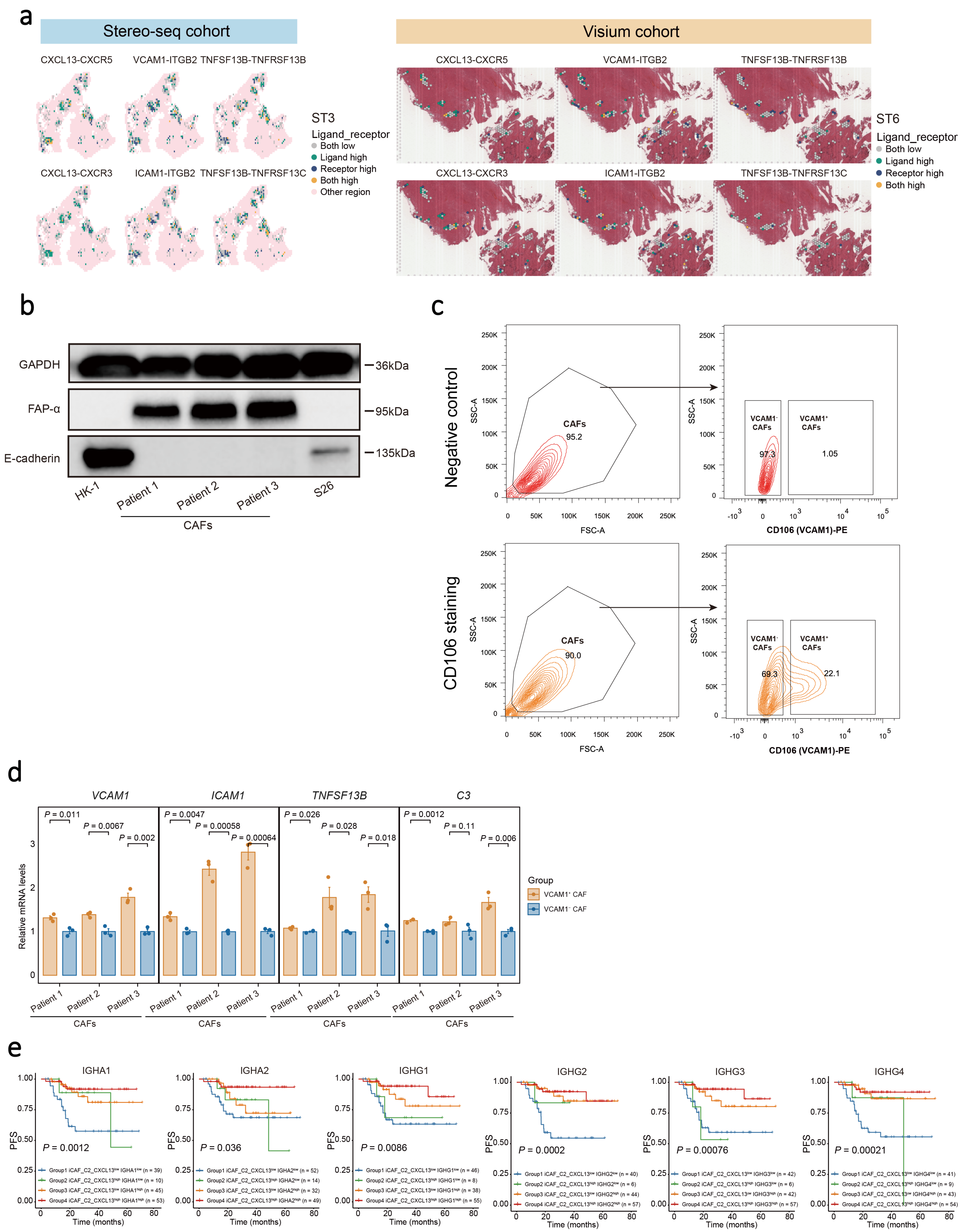
a
Stereo-seq cohort
Visium cohort
CXCL13-CXCR5
VCAM1-ITGB2
TNFSF13B-TNFRSF13B
CXCL13-CXCR3
ICAM1-ITGB2
TNFSF13B-TNFRSF13C
ST3
ST6
Ligand_receptor
Both low
Ligand high
Receptor high
Both high
Other region
b
GAPDH
FAP-α
E-cadherin
36kDa
95kDa
135kDa
HK-1
Patient 1
Patient 2
Patient 3
S26
CAFs
c
Negative control
CD106 staining
SSC-A
FSC-A
CAFs
95.2
90.0
VCAM1⁻ CAFs
VCAM1⁺ CAFs
97.3
1.05
69.3
22.1
CD106 (VCAM1)-PE
d
VCAM1
ICAM1
TNFSF13B
C3
Relative mRNA levels
P = 0.011
P = 0.0067
P = 0.002
P = 0.0047
P = 0.00058
P = 0.00064
P = 0.026
P = 0.028
P = 0.018
P = 0.0012
P = 0.11
P = 0.006
Group
VCAM1⁺ CAF
VCAM1⁻ CAF
e
IGHA1
IGHA2
IGHG1
IGHG2
IGHG3
IGHG4
PFS
Time (months)
P = 0.0012
P = 0.036
P = 0.0086
P = 0.0002
P = 0.00076
P = 0.00021

Supplementary Fig. 12 Functional characteristics and prognostic value of iCAF_C2_CXCL13 CAFs.

a Spatial distribution of cellular interactions in representative NPC section of samples from the Stereo-seq (left) and the Visium (right) cohorts. Ligand-receptor pairs are indicated on top, with expression level indicators at the right panel.

b Western blotting assay showing the protein expression of GAPDH, E-cadherin (epithelial cell marker), and FAP-α (fibroblast marker) in NPC cell lines (HK-1 and S26) and CAFs (patient 1, patient 2, and patient 3) derived from NPC tissues.

c Representative image of flow cytometry analysis for negative control (top) and CD106 (VCAM1) staining (bottom) CAFs in NPC biopsy tissue samples. Cells were stained with a VCAM1 antibody.

d Bar plots showing the relative expression levels of *VCAM1*, *ICAM1*, *TNFSF13B*, and *C3* in $VCAM1^{+}$ and $VCAM1^{-}$ CAFs (n = 3). P values are derived from two-sided student t-tests. The data is presented as mean ± SD.

e Kaplan-Meier survival curves of NPC patients (n = 147) stratified by combining the expression of IgH genes and the proportion of iCAF_C2_CXCL13 CAFs in the Bulk-RNA-seq cohort. Survival duration and probability were indicated at the x- and y-axis, respectively. *P* value and HR were calculated using two-sided cox tests. PFS, progression-free survival.

Source data are provided as a Source Data file.

sFigure 13

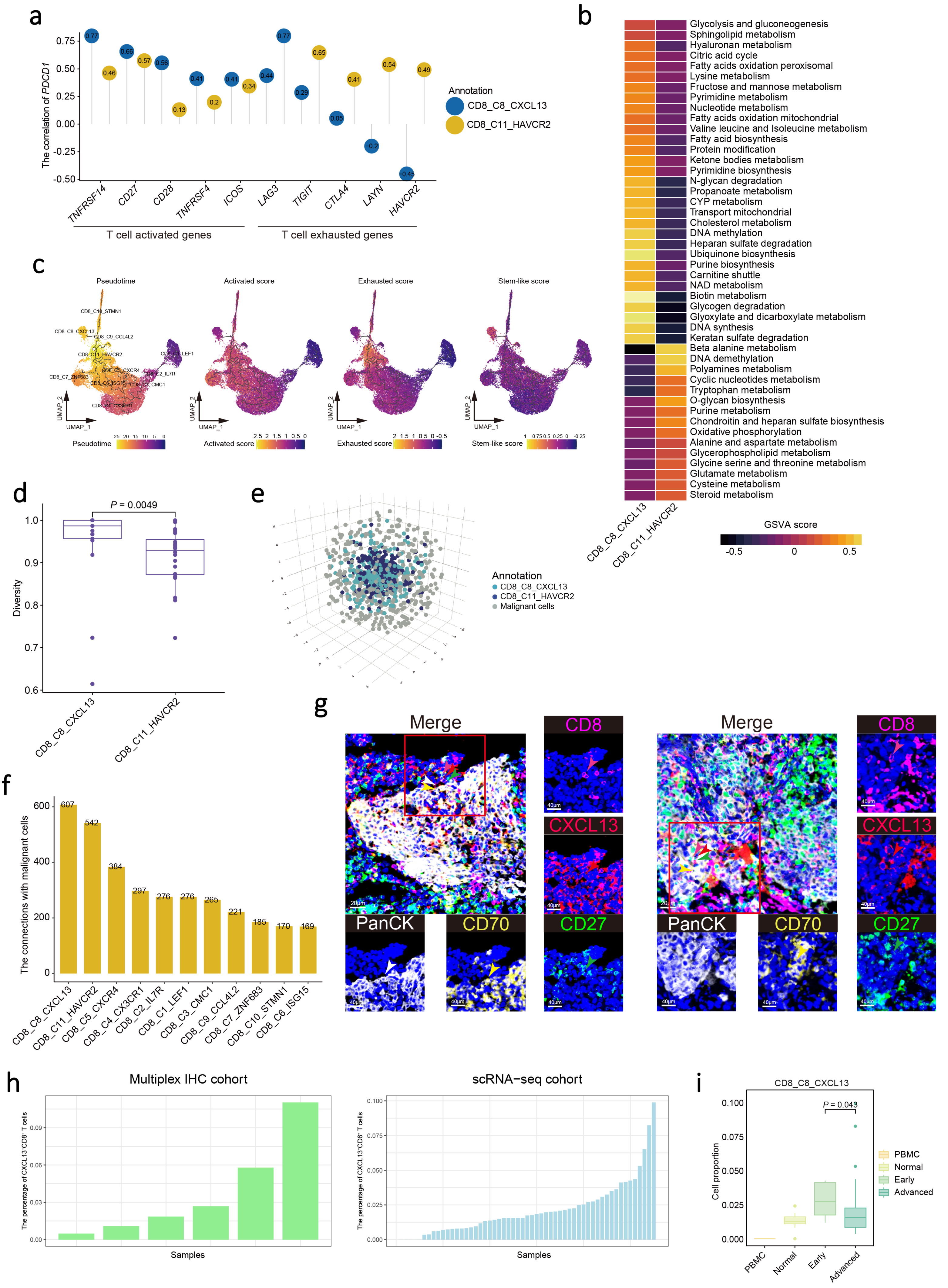

a
b
c
d
e
f
g
h
i
The correlation of PDCD1
TNFRSF14
CD27
CD28
TNFRSF4
ICOS
LAG3
TIGIT
CTLA4
LAYN
HAVCR2
T cell activated genes
T cell exhausted genes
Annotation
CD8_C8_CXCL13
CD8_C11_HAVCR2
Glycolysis and gluconeogenesis
Sphingolipid metabolism
Hyaluronan metabolism
Citric acid cycle
Fatty acids oxidation peroxisomal
Lysine metabolism
Fructose and mannose metabolism
Pyrimidine metabolism
Nucleotide metabolism
Fatty acids oxidation mitochondrial
Valine leucine and Isoleucine metabolism
Fatty acid biosynthesis
Protein modification
Ketone bodies metabolism
Pyrimidine biosynthesis
N-glycan degradation
Propanoate metabolism
CYP metabolism
Transport mitochondrial
Cholesterol metabolism
DNA methylation
Heparan sulfate degradation
Ubiquinone biosynthesis
Purine biosynthesis
Carnitine shuttle
NAD metabolism
Biotin metabolism
Glycogen degradation
Glyoxylate and dicarboxylate metabolism
DNA synthesis
Keratan sulfate degradation
Beta alanine metabolism
DNA demethylation
Polyamines metabolism
Cyclic nucleotides metabolism
Tryptophan metabolism
O-glycan biosynthesis
Purine metabolism
Chondroitin and heparan sulfate biosynthesis
Oxidative phosphorylation
Alanine and aspartate metabolism
Glycerophospholipid metabolism
Glycine serine and threonine metabolism
Glutamate metabolism
Cysteine metabolism
Steroid metabolism
GSVA score
Pseudotime
Activated score
Exhausted score
Stem-like score
UMAP_1
UMAP_2
P = 0.0049
Diversity
Malignant cells
The connections with malignant cells
CD8_C5_CXCR4
CD8_C4_CX3CR1
CD8_C2_IL7R
CD8_C1_LEF1
CD8_C3_CMC1
CD8_C9_CCL4L2
CD8_C7_ZNF683
CD8_C10_STMN1
CD8_C6_ISG15
Merge
CD8
CXCL13
PanCK
CD70
CD27
Multiplex IHC cohort
scRNA−seq cohort
Samples
The percentage of CXCL13+CD8+ T cells
Cell proportion
P = 0.043
PBMC
Normal
Early
Advanced

Supplementary Fig. 13 Transcriptional and developmental features of $CD8^+$ T cells.

a Dot plots showing the correlation between PDCD1 and the activated (left) and exhausted (right) related genes in CD8_C8_CXCL13 (blue) and CD8_C11_HAVCR2 (yellow) cells. Genes of interest are presented at the x-axis, and Pearson correlation coefficients are presented at the y-axis.

b Heatmap showing GSVA scores of the gene signatures relevant to metabolism and epigenetic modification in CD8_C8_CXCL13 and CD8_C11_HAVCR2 cells. Each specific pathway (row) was coloured dark blue to yellow for low to high GSVA scores.

c Trajectory UMAP plots of $CD8^+$ T cells (n = 41,736) showing pseudotime, activated, exhausted, and stem-like signature scores for each cell (dot) with colours from dark blue to yellow for early to terminal states.

d Box plots showing the TCR diversity for CD8_C8_CXCL13 and CD8_C9_HAVCR2 cells (n = 20). Clusters and TCR diversity scores are indicated at the x- and y-axis, respectively. Endpoints depict minimum and maximum values; centre lines denote median values; whiskers denote 1.5 × the interquartile range; coloured dots denote each patient. The comparison was made using a paired two-sided student t-test.

e 3D visualization of interactions between malignant cells and CD8_C8_CXCL13 or CD8_C11_HAVCR2 in NPC. Interactions were predicted using the CSOmap program.

f Bar plots showing the number of connections between malignant cells and $CD8^+$ T cell clusters in NPC.

g Representative images of multiplex IHC staining for the juxtaposition of CD70-expressing malignant cells ($PanCK^+$) and CD27-expressing $CXCL13^+CD8^+$ T cells ($CXCL13^+CD8^+$) in NPC tissue samples. Proteins detected using respective antibodies are indicated on top. The purple, red, green, yellow, and white arrows indicated positive cells with the expression of CD8, CXCL13, CD27, CD70, and PanCK proteins in NPC tissue, respectively (bottom panel). Images are representative of three independent samples. Scale bars are 20μm or 40μm, respectively.

h Bar plot showing the percentage of $CXCL13^+CD8^+$ T cells for multiplex IHC (n = 6; left panel) and scRNA-seq (n = 55; right panel) cohort.

i Box plots showing the proportion of $CD8^+$ T cell clusters among all $CD8^+$ T cells in samples of different sources and clinical stages of NPC (n = 68, remove samples where malignant cells were not detected in the NPC tissue and samples with fewer than 100 $CD8^+$ T cells). *P* values are derived from two-sided student t-tests.

In box plots, endpoints depict minimum and maximum values; centre lines denote median values; whiskers denote 1.5 × the interquartile range. Source data are provided as a Source Data file.

sFigure 14

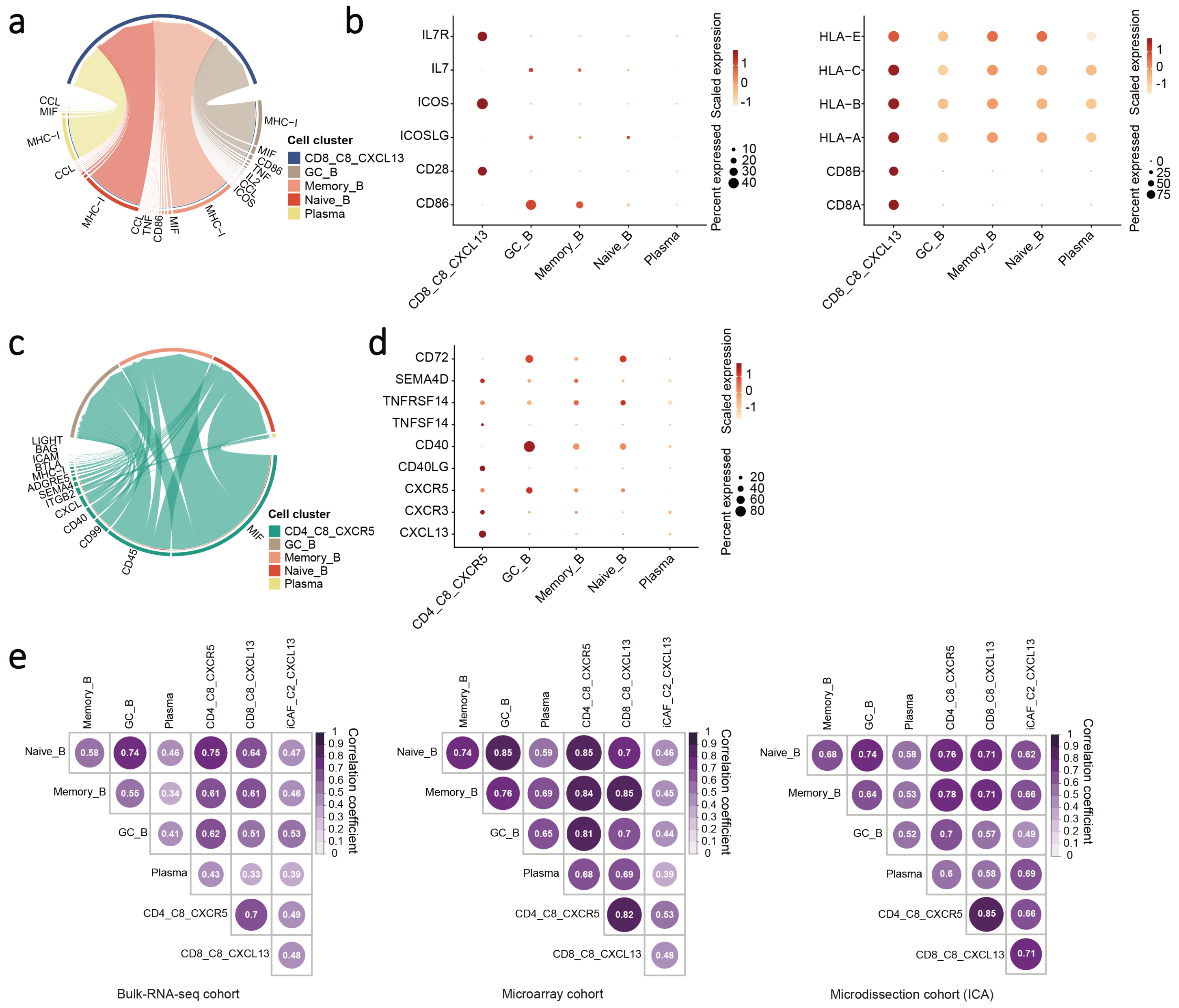

a
CCL
MIF
MHC−I
CCL
MHC−I
CCL
TNF
CD86
MIF
MHC−I
MHC−I
MIF
CD86
TNF
CCL
ICOS
Cell cluster
CD8_C8_CXCL13
GC_B
Memory_B
Naive_B
Plasma
b
IL7R
IL7
ICOS
ICOSLG
CD28
CD86
CD8_C8_CXCL13
GC_B
Memory_B
Naive_B
Plasma
Scaled expression
1
0
-1
Percent expressed
10
20
30
40
HLA−E
HLA−C
HLA−B
HLA−A
CD8B
CD8A
CD8_C8_CXCL13
GC_B
Memory_B
Naive_B
Plasma
Scaled expression
1
0
-1
Percent expressed
0
25
50
75
c
LIGHT
BAG
ICAM
BTLA
MHC−I
ADGRE5
SEMA4
ITGB2
CXCL
CD40
CD99
CD45
MIF
Cell cluster
CD4_C8_CXCR5
GC_B
Memory_B
Naive_B
Plasma
d
CD72
SEMA4D
TNFRSF14
TNFSF14
CD40
CD40LG
CXCR5
CXCR3
CXCL13
CD4_C8_CXCR5
GC_B
Memory_B
Naive_B
Plasma
Scaled expression
1
0
-1
Percent expressed
20
40
60
80
e
Memory_B
GC_B
Plasma
CD4_C8_CXCR5
CD8_C8_CXCL13
iCAF_C2_CXCL13
Naive_B 0.58 0.74 0.46 0.75 0.64 0.47
Memory_B 0.55 0.34 0.61 0.61 0.46
GC_B 0.41 0.62 0.51 0.53
Plasma 0.43 0.33 0.39
CD4_C8_CXCR5 0.7 0.49
CD8_C8_CXCL13 0.48
Correlation coefficient
1
0.9
0.8
0.7
0.6
0.5
0.4
0.3
0.2
0.1
0
Bulk-RNA-seq cohort
Memory_B
GC_B
Plasma
CD4_C8_CXCR5
CD8_C8_CXCL13
iCAF_C2_CXCL13
Naive_B 0.74 0.85 0.59 0.85 0.7 0.46
Memory_B 0.76 0.69 0.84 0.85 0.45
GC_B 0.65 0.81 0.7 0.44
Plasma 0.68 0.69 0.39
CD4_C8_CXCR5 0.82 0.53
CD8_C8_CXCL13 0.48
Correlation coefficient
Microarray cohort
Memory_B
GC_B
Plasma
CD4_C8_CXCR5
CD8_C8_CXCL13
iCAF_C2_CXCL13
Naive_B 0.68 0.74 0.58 0.76 0.71 0.62
Memory_B 0.64 0.53 0.78 0.71 0.66
GC_B 0.52 0.7 0.57 0.49
Plasma 0.6 0.58 0.69
CD4_C8_CXCR5 0.85 0.66
CD8_C8_CXCL13 0.71
Correlation coefficient
Microdissection cohort (ICA)

Supplementary Fig. 14 Intensive interactions among TLS-associated cell clusters.

a Chord diagrams showing the ligand-receptor interactions between CD4_C8_CXCR5 and B lineage cells (Naïve_B, Memory_B, GC_B, and plasma cells) in NPC. The different colour bars around chord diagrams represented different clusters. Ribbons connecting chains indicate the frequency of the interactions, coloured according to clusters.

b Dot plots showing the expression levels of ligand-receptor genes for CD4_C8_CXCR5 and B lineage cells (Naïve_B, Memory_B, GC_B, and plasma cells) in NPC. The scaled expression levels of molecules are indicated by a colour heatmap. The percentage of cells expressing the molecule in a cluster is indicated by circle size.

c Chord diagrams showing the ligand-receptor interactions between CD8_C8_CXCL13 and B lineage cells (Naïve_B, Memory_B, GC_B, and plasma cells) in NPC. The different colour bars around chord diagrams represented different clusters. Ribbons connecting chains indicate the frequency of the interactions, coloured according to clusters.

d Dot plots showing the expression levels of ligand-receptor genes for CD8_C8_CXCL13 and B lineage cells (Naïve_B, Memory_B, GC_B, and plasma cells) in NPC. The scaled expression levels of molecules are indicated by a colour heatmap. The percentage of cells expressing the molecule in a cluster is indicated by circle size.

e Dot plots showing the pairwise correlation of TLS-associated cell clusters in the Bulk-RNA-seq (n = 147; left panel), the Microarray (n = 150; middle panel), and the Microdissection (n = 46; right panel) cohorts. The correlation was assessed using Pearson’s correlation test and indicated as heatmap colours and circle sizes.

sFigure 15

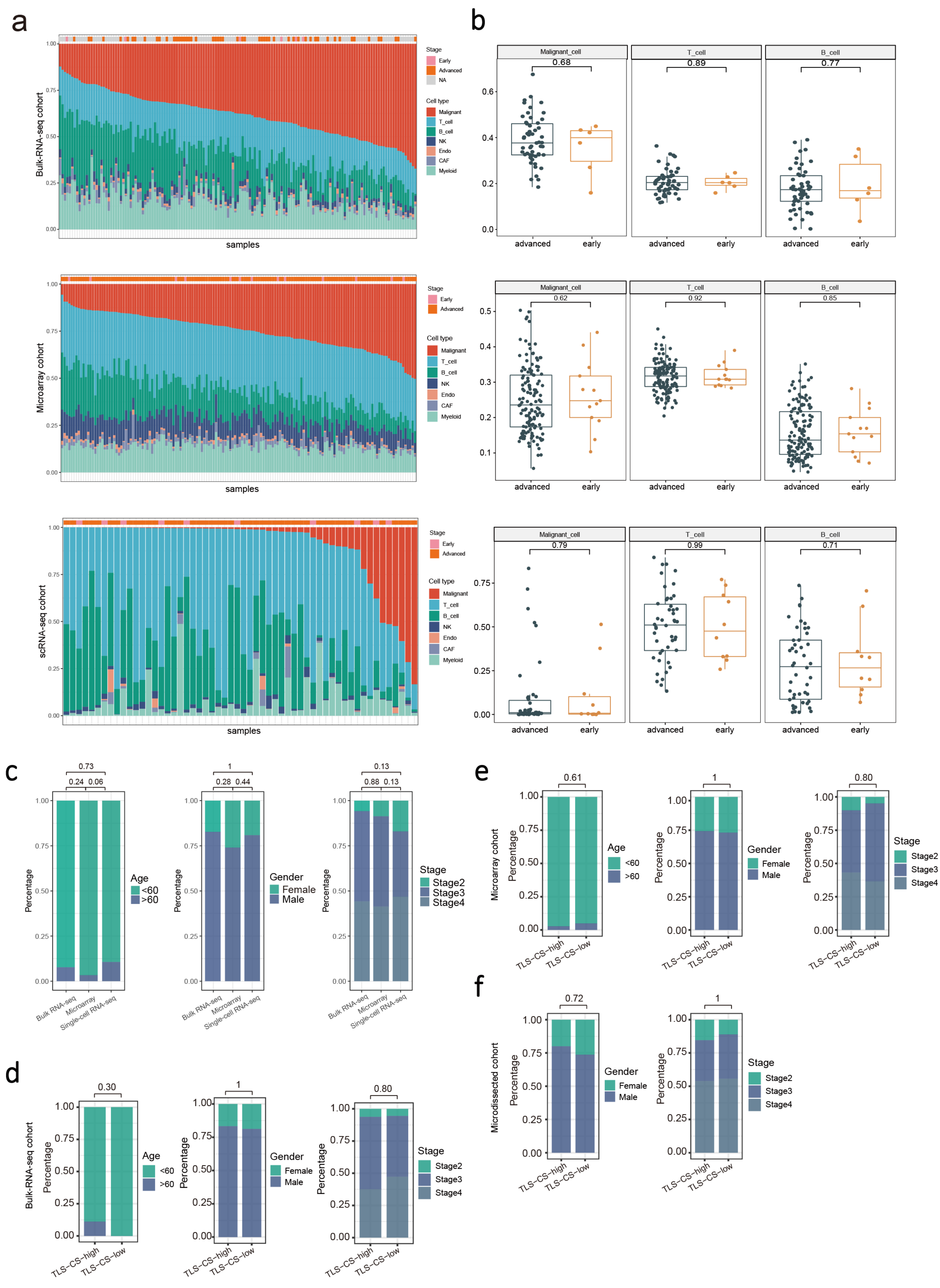
a
b
c
d
e
f
Bulk-RNA-seq cohort
Microarray cohort
scRNA-seq cohort
Microdissected cohort
samples
Stage
Early
Advanced
NA
Cell type
Malignant
T_cell
B_cell
NK
Endo
CAF
Myeloid
Malignant_cell
T_cell
B_cell
advanced
early
Percentage
Age
<60
>60
Gender
Female
Male
Stage2
Stage3
Stage4
Bulk RNA-seq
Microarray
Single-cell RNA-seq
TLS-CS-high
TLS-CS-low

Supplementary Fig. 15 Additional characterise of NPC cohorts

a Bar plots showing the cell proportions (y-axis) of each cell types for each sample (x-axis) in Bulk RNA-seq (n = 147; top panel), Microarray (n = 150; middle panel), and Single-cell RNA-seq (n = 56; bottom panel) cohorts. Cell types are colour-coded and indicated on the right.

b Box plots showing the T, B, and tumour cell signature in samples from the Bulk RNA-seq (n = 147; top panel), Microarray (n = 150; middle panel), and Single-cell RNA-seq (n = 56; bottom panel) cohorts, respectively.

c Bar plots showing the proportion of different age (left panel), gender (middle panel), and stage (right panel) groups in Bulk RNA-seq (n = 52), Microarray (n = 150), and Single-cell RNA-seq (n = 47) cohorts. Chip-square test was performed to evaluate the significant difference. NS: not significant (*P* value > 0.05).

d-f Bar plots showing the sample proportions for different age (left panel), gender (middle panel), and clinical stage (right panel) categories between the TLS-CS-high and TLS-CS-low groups for the Bulk RNA-seq (n = 52; **d**), Microarray (n = 150; **e**), and Microdissction cohorts (n = 44; **f**). Chip-square test was performed to evaluate the significant difference.

In box plots, endpoints depict minimum and maximum values; centre lines denote median values; whiskers denote 1.5 × the interquartile range. Source data are provided as a Source Data file.

sFigure 16

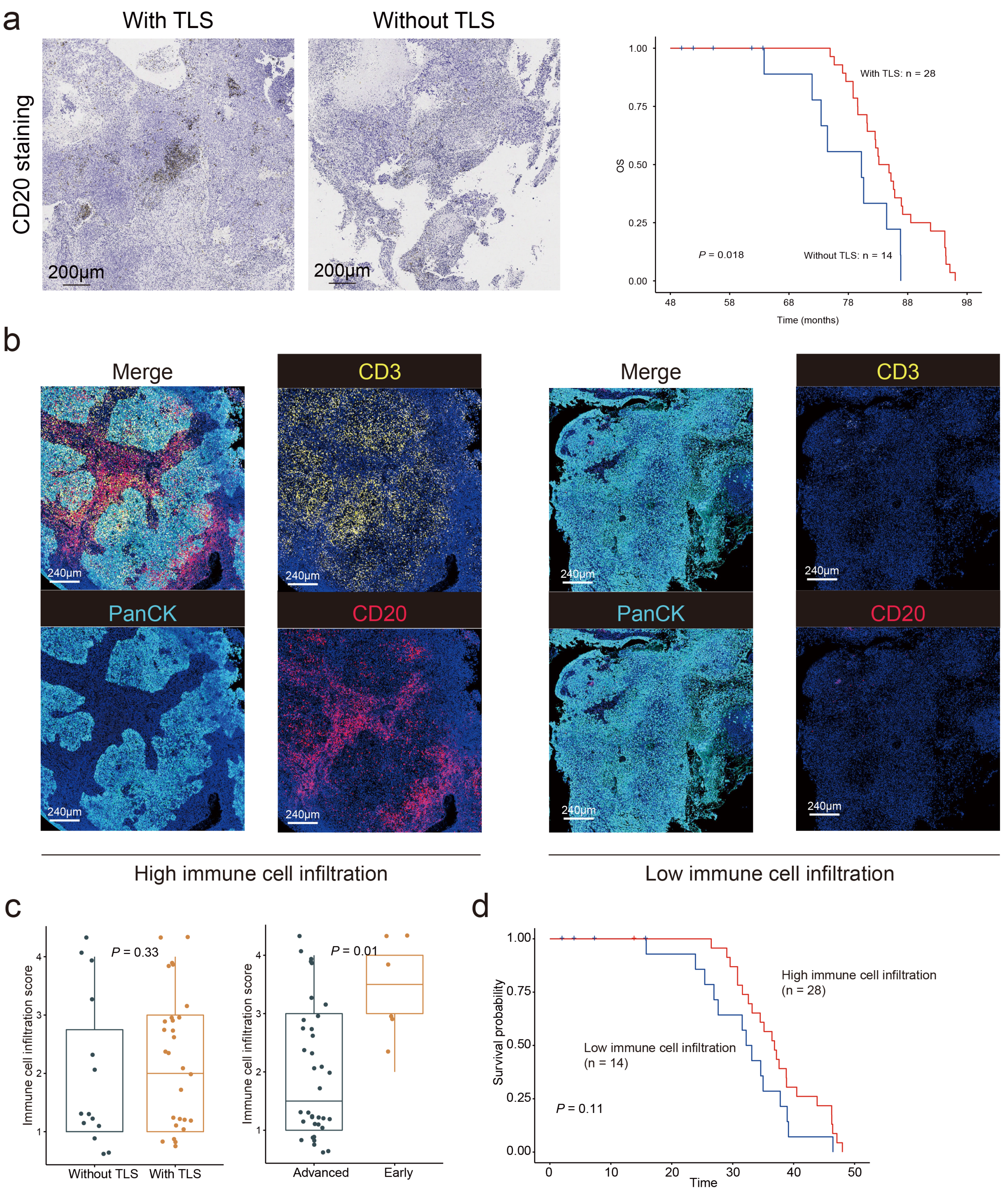
a
With TLS
Without TLS
CD20 staining
200µm
200µm
1.00
0.75
0.50
0.25
0.00
OS
With TLS: n = 28
Without TLS: n = 14
P = 0.018
48
58
68
78
88
98
Time (months)
b
Merge
CD3
PanCK
CD20
240µm
High immune cell infiltration
Low immune cell infiltration
c
Immune cell infiltration score
P = 0.33
Without TLS
With TLS
P = 0.01
Advanced
Early
d
Survival probability
High immune cell infiltration
(n = 28)
Low immune cell infiltration
(n = 14)
P = 0.11
0
10
20
30
40
50
Time

Supplementary Fig. 16 The relationship between TLS and tumour infiltrating immune cells in NPC.

a IHC staining of CD20 in NPC tumour samples. CD20 is considered a marker of TLS. Scale bars are 200um. Kaplan-Meier survival curves of NPC cohorts (n = 42) with patients stratified by presence of TLS in tumour section. Survival duration and probability are indicated at the x- and y-axis, respectively. *P* value and HR were calculated using a two-sided cox test. OS, overall survival.

b Multiplex IHC staining of T, B, and malignant cells in NPC tissue biopsy. Cells were coloured according to their staining with CD3 (yellow), CD20 (red), and PanCK (cyan) proteins as indicated on top. Images are representative of three independent samples. Scale bar is 240μm.

c Box plots showing the immune cell infiltration scores in samples with or without TLS (n = 42, left panel) and at advanced or early stage (right panel), respectively.

d Kaplan-Meier survival curves of NPC cohorts (n = 42) with patients stratified by immune cell infiltration in tumour section. Survival duration and probability are indicated at the x- and y-axis, respectively. *P* value and HR were calculated using a two-sided cox test. OS, overall survival.

In box plots, endpoints depict minimum and maximum values; centre lines denote median values; whiskers denote 1.5 × the interquartile range. Source data are provided as a Source Data file.

sFigure 17

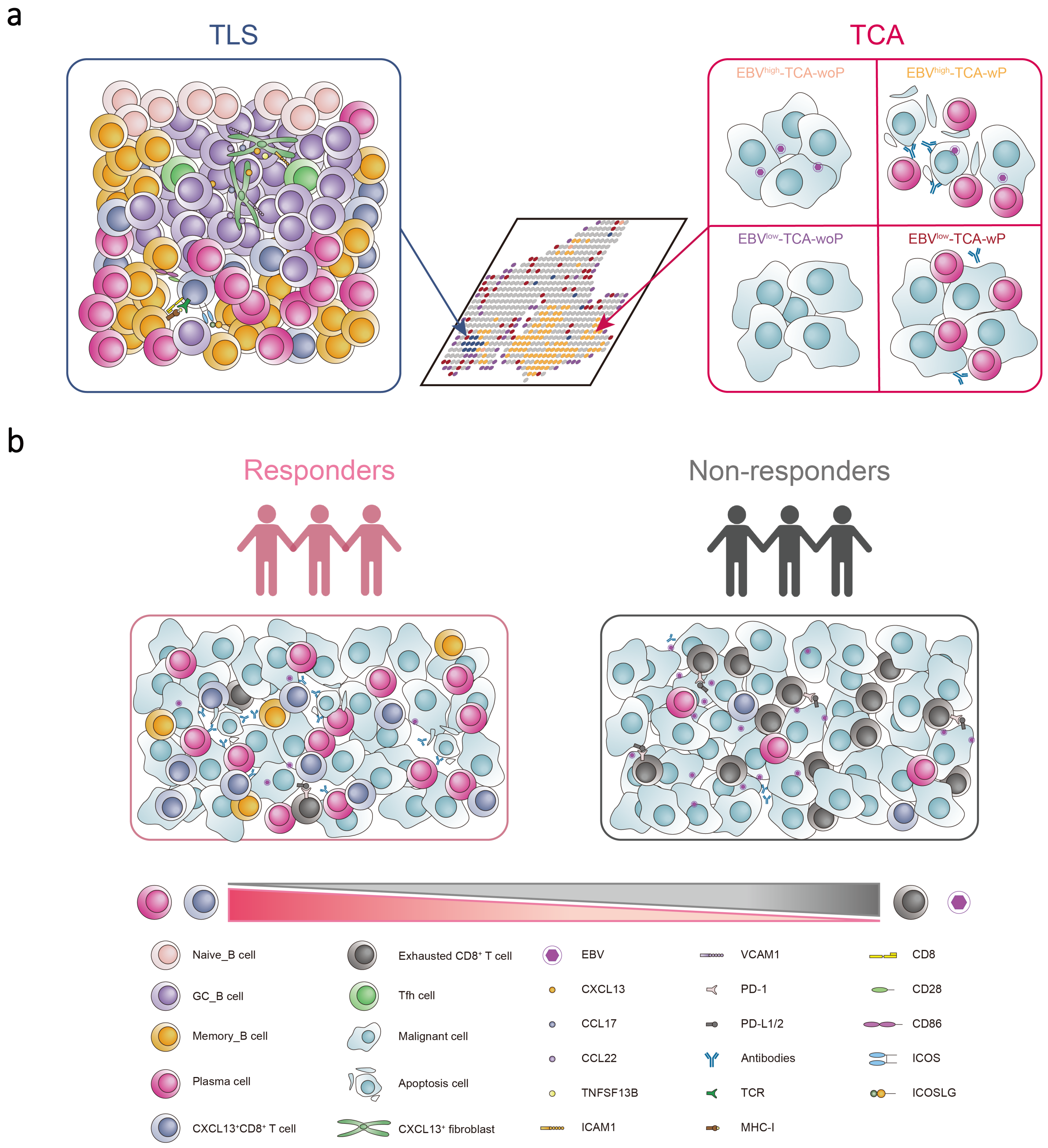
a
TLS
TCA
EBV^high-TCA-woP
EBV^high-TCA-wP
EBV^low-TCA-woP
EBV^low-TCA-wP
b
Responders
Non-responders
Naive_B cell
GC_B cell
Memory_B cell
Plasma cell
CXCL13+CD8+ T cell
Exhausted CD8+ T cell
Tfh cell
Malignant cell
Apoptosis cell
CXCL13+ fibroblast
EBV
CXCL13
CCL17
CCL22
TNFSF13B
ICAM1
VCAM1
PD-1
PD-L1/2
Antibodies
TCR
MHC-I
CD8
CD28
CD86
ICOS
ICOSLG

Supplementary Fig. 17 Schematic diagram of cellular composition and interactions of TLS and TCA in NPC.

a Our study dissects the spatial localisation and composition of TLS and TCA in NPC at single-cell and spatial resolutions. Essential cellular components of TLS in NPC include Naïve_B, Memory_B, GC_B, plasma cells, $CD4^+$ T follicular helper (Tfh) cells, $CXCL13^+$ $CD8^+$ T cells, and $CXCL13^+$ CAFs. Naïve B cells migrate to TLS and differentiate into antibody-secreting plasma cells through GC reactions. Within TLS, $CXCL13^+$ CAFs secrete chemokines (such as CXCL13, CCL17, and CCL22) to recruit multiple immune cells, promote B cell residency through adhesion molecules (such as VCAM1 and ICAM1), and support B cell maturation and antibody production through ligands and cytokines (such as TNFSF13B). B cells also act as antigen-presenting cells and activate $CXCL13^+$ $CD8^+$ T cells through MHC-CD8, ICOSLG-ICOS, and CD86-CD28 ligand-receptor pairs. We divide TCAs into four types, including EBV^{high}-TCA-wP, EBV^{high} -TCA-woP, EBV^{low}-TCA-wP, and EBV^{low}-TCA-woP. Among all TCAs, EBV^{high}-TCA-wP exhibits the highest apoptosis feature, where tumour-infiltrating plasma cells secrete antibodies recognizing EBV infection-related antigens and promote the apoptosis of EBV high malignant cells.

b Integrative analysis of multi-omics data reveals the differences in the TME between the responders and non-responders of NPC patients who received ICB. Patients are categorized into responders and non-responders based on their response to ICB (top). Responders have a higher proportion of plasma cells that secrete antibodies recognizing EBV infection-related antigens, promoting the apoptosis of EBV^{high} malignant cells, and a higher proportion of $CXCL13^+CD8^+$ T cells as effector T cells for the anti-tumour response, compared to non-responders (middle). By contrast, the non-responders have a higher EBV load and a more profound immune-suppressive tumour microenvironment due to the lack of plasma cells and $CXCL13^+CD8^+$ T cells, which results in more exhausted $CD8^+$ T cells (middle).

Cells and molecules in (**a**) and (**b**) are annotated in the bottom panel of (**b**). TCA, tumour cell aggregates; EBV^{high}-TCA-wP, EBV^{high}-TCA with plasma cells; EBV^{low}-TCA-wP, EBV^{low}-TCA with plasma cells; EBV^{high}-TCA-woP, EBV^{high}-TCA without plasma cells; EBV^{low}-TCA-woP, EBV^{low}-TCA without plasma cells. The diagrams of antibody and human models were created with BioRender.com released under a Creative Commons Attribution-NonCommercial-NoDerivs 4.0 International license.
