# Supplementary material for: Single-cell and spatial transcriptome analyses reveal tertiary lymphoid structures linked to tumour progression and immunotherapy response in nasopharyngeal carcinoma
Source: Nat Commun. 2024 Sep 4;15:7713. doi: 10.1038/s41467-024-52153-4 (PMC11375053; doi:10.1038/s41467-024-52153-4)
Supplement: Supplementary file 3 — Description of Additional Supplementary Files [file 41467_2024_52153_MOESM3_ESM.pdf]

Supplementary Data 1
Clinical characteristics of NPC cohorts

Supplementary Data 2
Differentially expressed genes in different cell clusters of NPC

Supplementary Data 3
BCR heavy chain information of B cells in NPC

Supplementary Data 4
Signature score related genes

Supplementary Data 5
TCR information of T cells in NPC

Supplementary Data 6
Prognostic value of TLS in the microarray and Bulk-RNA-seq NPC sample collections.

Supplementary Data 7
Primer-id sequence (5'-3')
