# Supplementary material for: Single-cell and spatial transcriptome analyses reveal tertiary lymphoid structures linked to tumour progression and immunotherapy response in nasopharyngeal carcinoma
Source: Nat Commun. 2024 Sep 4;15:7713. doi: 10.1038/s41467-024-52153-4 (PMC11375053; doi:10.1038/s41467-024-52153-4)
Supplement: Supplementary file 11 — Reporting Summary [file 41467_2024_52153_MOESM11_ESM.pdf]

nature portfolio

Corresponding author(s): Jin-Xin Bei

Last updated by author(s): Jun 28, 2024

Reporting Summary

Nature Portfolio wishes to improve the reproducibility of the work that we publish. This form provides structure for consistency and transparency in reporting. For further information on Nature Portfolio policies, see our Editorial Policies and the Editorial Policy Checklist.

Statistics

For all statistical analyses, confirm that the following items are present in the figure legend, table legend, main text, or Methods section.

n/a	Confirmed	
☐	☒	The exact sample size (*n*) for each experimental group/condition, given as a discrete number and unit of measurement
☐	☒	A statement on whether measurements were taken from distinct samples or whether the same sample was measured repeatedly
☐	☒	The statistical test(s) used AND whether they are one- or two-sided<br>*Only common tests should be described solely by name; describe more complex techniques in the Methods section.*
☒	☐	A description of all covariates tested
☐	☒	A description of any assumptions or corrections, such as tests of normality and adjustment for multiple comparisons
☐	☒	A full description of the statistical parameters including central tendency (e.g. means) or other basic estimates (e.g. regression coefficient) AND variation (e.g. standard deviation) or associated estimates of uncertainty (e.g. confidence intervals)
☐	☒	For null hypothesis testing, the test statistic (e.g. *F*, *t*, *r*) with confidence intervals, effect sizes, degrees of freedom and *P* value noted<br>*Give P values as exact values whenever suitable.*
☒	☐	For Bayesian analysis, information on the choice of priors and Markov chain Monte Carlo settings
☒	☐	For hierarchical and complex designs, identification of the appropriate level for tests and full reporting of outcomes
☐	☒	Estimates of effect sizes (e.g. Cohen's *d*, Pearson's *r*), indicating how they were calculated

Our web collection on statistics for biologists contains articles on many of the points above.

Software and code

Policy information about availability of computer code

Data collection	Illumina HiSeq and NovaSeq system, and MGI DNBSEQ sequencer to generate sequencing data; FACS Arial III to generate Flow cytometry data.
Data analysis	Flow cytometry data was analysed using BD FACS Diva version 8.<br>Transcriptome data was analysed using bcl2fastq (https://support.illumina.com/sequencing/sequencing_software/bcl2fastq-conversion-software/downloads.html; version 2.19.0.316), CellRanger (http://10xgenomics.com/; version 3.0.1), Seurat (https://satijalab.org/Seurat; version 4.0.0), DoubletFinder (https://github.com/chris-mcginnis-ucsf/DoubletFinder; version 2.0.1), Harmony (https://github.com/immunogenomics/harmony; version 1.0), Space Ranger (http://10xgenomics.com/; version 1.3.0), SAW (https://github.com/BGIResearch/SAW; version 5.5.0), SpaGene (https://github.com/liuqivandy/SpaGene; version 0.1.0), BayesSpace (https://github.com/edward130603/BayesSpace; version 1.0.0), RSEM (https://github.com/deweylab/RSEM; version 1.3.3), STAR (https://github.com/alexdobin/STAR; version 2.6.1), Command Console Software (https://www.thermofisher.cn/cn/zh/home/life-science/microarray-analysis.html; version 4.0), Transcriptome Analysis Console (https://www.thermofisher.cn/cn/zh/home/life-science/microarray-analysis.html; version 4.0.1), ssGSEA (https://github.com/broadinstitute/ssGSEA2.0; version 2.0), ChangeO (https://changeo.readthedocs.io/en/stable/install.html; version 1.0.0), IgBLAST (ftp://ftp.ncbi.nih.gov/blast/executables/igblast/release/LATEST; version 1.17.0), SHazaM (https://shazam.readthedocs.io/en/stable/; version 1.0.2), Monocle3 (http://cole-trapnell-lab.github.io/monocle3/; version 0.0.2), STARTRAC (https://github.com/Japrin/STARTRAC; version 0.1), GSVA (https://github.com/rcastelo/GSVA; version 1.30.0), ClusterProfiler (https://github.com/YuLab-SMU/clusterProfiler; version 4.0), Diffusion maps (https://github.com/theislab/destiny) CSOmap (https://github.com/lijxug/CSOmapR; version 1.0), CellChat (https://github.com/sqjin/CellChat; version 1.0.0), ggplot2 (https://cran.r-project.org/web/packages/ggplot2/; version 3.3.3), online tool CIBERSORTx (https://cibersortx.stanford.edu/) . In-house scripts are available at https://github.com/yliuup/NPC-TLS.

For manuscripts utilizing custom algorithms or software that are central to the research but not yet described in published literature, software must be made available to editors and reviewers. We strongly encourage code deposition in a community repository (e.g. GitHub). See the Nature Portfolio guidelines for submitting code & software for further information.

Data

Policy information about availability of data

All manuscripts must include a data availability statement. This statement should provide the following information, where applicable:

- Accession codes, unique identifiers, or web links for publicly available datasets
- A description of any restrictions on data availability
- For clinical datasets or third party data, please ensure that the statement adheres to our policy

The raw sequence data (BCR and spatial data) generated in this study have been deposited in the Genome Sequence Archive (GSA) of the National Genomics Data Center (NGDC), Beijing Institute of Genomics (China National Center for Bioinformation), Chinese Academy of Sciences, under accession number HRA006885 (https://ngdc.cncb.ac.cn/gsa-human/browse/HRA006885). The raw data are available under controlled access due to data privacy laws related to patient consent for data sharing. The data should be used for research purposes only. According to the guidelines of GSA-human, all non-profit researchers are allowed access to the data, and the Principal Investigator of any research group can apply for the data following the guidelines at the GSA database portal (https://ngdc.cncb.ac.cn/gsa-human/). The response time for access requests is approximately 10 working days. Once access has been granted, the data will be available for download within one month. The user can also contact the corresponding author directly for inquiries.

This study's processed single-cell VDJ-seq and spatial transcriptomic data can be obtained from Gene Expression Omnibus (GEO) with an accession number of GSE206245 and the Research Data Deposit (RDD; http://www.researchdata.org.cn/) with an accession number of RDDB2024955995. The NPC single-cell RNA and bulk RNA-seq publicly available data used in this study are available in the GEO database under accession code GSE162025 (https://www.ncbi.nlm.nih.gov/geo/query/acc.cgi?&acc=GSE162025), GSE150825 (https://www.ncbi.nlm.nih.gov/geo/query/acc.cgi?&acc=GSE150825), GSE150430 (https://www.ncbi.nlm.nih.gov/geo/query/acc.cgi?&acc=GSE150430), GSE102349 (https://www.ncbi.nlm.nih.gov/geo/query/acc.cgi?&acc=GSE102349), GSE121600 (https://www.ncbi.nlm.nih.gov/geo/query/acc.cgi?acc=GSE121600), GSA database under accession code HRA000087 (https://ngdc.cncb.ac.cn/gsa-human/browse/HRA000087) and the URL https://www.science.org/doi/10.1126/sciadv.abh2445. GaC Bulk mRNA-seq expression data (normalized) generated by The Cancer Genome Atlas (TCGA) on primary stomach adenocarcinoma were downloaded from NCI Cancer Genomic Data Commons (NCI-GDC: https://gdc.cancer.gov). The remaining data are available within the Article, Supplementary Information, Source Data file or available from the authors upon request. Source data are provided with this paper.

Research involving human participants, their data, or biological material

Policy information about studies with human participants or human data. See also policy information about sex, gender (identity/presentation), and sexual orientation and race, ethnicity and racism.

Reporting on sex and gender	The sex and gender were not considered in study design.
Reporting on race, ethnicity, or other socially relevant groupings	The race, ethnicity, and other socially relevant groupings were not considered in study design.
Population characteristics	All patients with pathological NPC and EBV+ GaC diagnosis were enrolled in this study. Detailed information can be found in the Patient recruitment and sample collection section of Methods and Supplementary Data 1.
Recruitment	The donors are recruited from independent cohorts, avoiding the selection of poorly clinically characterized volunteers.
Ethics oversight	Nasopharyngeal carcinoma and ebv+ gastric cancer patients; The medical ethics committee of the Institutional Review Board of Sun Yat-sen University Cancer Centre.

Note that full information on the approval of the study protocol must also be provided in the manuscript.

Field-specific reporting

Please select the one below that is the best fit for your research. If you are not sure, read the appropriate sections before making your selection.

☒ Life sciences ☐ Behavioural & social sciences ☐ Ecological, evolutionary & environmental sciences

For a reference copy of the document with all sections, see nature.com/documents/nr-reporting-summary-flat.pdf

Life sciences study design

All studies must disclose on these points even when the disclosure is negative.

Sample size	Sample size for scRNA-seq and ST was determined by the availability of patient samples.<br>No statistical tests were performed for sample size calculation but it was sufficient for this proof-of-concept study. The exact number of samples used per figure is informed in each figure.
Data exclusions	All criteria for data exclusion were pre-established. We removed doublets in each sample by R package "DoubletFinder", with an expected doublet rate of 0.05 and default parameters used otherwise. Next, any cells were removed for which had either less than 101 UMIs, or expression of less than 501 genes, or over 15% UMIs linked to mitochondrial genes.
Replication	FACS, multiplex IHC staining, and co-culture assays were confirmed in at least three biological replicates.

Randomization	The patients with nasopharyngeal carcinoma and EBV+ gastric cancer were recruited randomly in this study.
Blinding	Investigators were blinded to patient origin. Tumour biopsy was collected and sent for downstream procedures with coded "Sample ID".

Reporting for specific materials, systems and methods

We require information from authors about some types of materials, experimental systems and methods used in many studies. Here, indicate whether each material, system or method listed is relevant to your study. If you are not sure if a list item applies to your research, read the appropriate section before selecting a response.

Materials & experimental systems

n/a	Involved in the study	
☐	☒	Antibodies
☐	☒	Eukaryotic cell lines
☒	☐	Palaeontology and archaeology
☒	☐	Animals and other organisms
☒	☐	Clinical data
☒	☐	Dual use research of concern
☒	☐	Plants

Methods

n/a	Involved in the study	
☒	☐	ChIP-seq
☐	☒	Flow cytometry
☒	☐	MRI-based neuroimaging

Antibodies

Antibodies used	All antibodies were commercially purchased and included: anti-human CD20 (mouse; Abcam, Cat. no. ab9475, 1:50), anti-human CD4 (rabbit; ZSbio, Cat. no. ZA-0519), anti-human CD8A (mouse; ZSbio, Cat. no. TA802079), anti-human FAP-α (Abcam, Cat. no. ab207178, 1:200), anti-human CASP3 (rabbit; CST, Cat. no. 9664, 1:1000), anti-human CD3 (rabbit; ZSbio, Cat no. ZA-0503), anti-human CD68 (mouse; ZSbio, Cat no. TA802952), anti-human CD56 (mouse; ZSbio, Cat no. ZM-0057), anti-human PanCK (mouse; ZSbio, Cat no. ZM-0069), anti-human CXCR5 (rabbit; Abcam, Cat. no. ab254415, 1:1000), anti-human PD1 (mouse; ZSbio, Cat no. ZM-0381), anti-human TIM3 (rabbit; CST, Cat. no. 45208, 1:400), anti-human IgG (rabbit; ZSbio, Cat no. ZA-0448), anti-human IgA (rabbit; ZSbio, Cat no. ZA-0446), anti-human E-Cadherin (mouse; CST, Cat. no. CST14472), anti-human VCAM1(mouse; BioLegend, Cat. no. 305805), anti-rabbit IgG (goat; CST, Cat. no. 7074, 1:2000), anti-mouse IgG (horse; CST, Cat. no. 7076, 1:2000), anti-human GAPDH (mouse; ABclonal, Cat. no. AC002, 1:5000), anti-human CXCL13 (rabbit, NOVUS, Cat. no. NBP2-16041, 1:100), anti-CD70 (CST, Cat. no. 69209, 1:500), and anti-CD27 (proteintech, Cat. no. 66308-1-lg, 1:1000).
Validation	All the antibodies used in this study were commercial antibodies, with validation procedures described on the following sites of the manufacturers:<br>anti-human CD20 (mouse; Abcam; Cat. no. ab9475)<br>https://www.abcam.com/cd20-antibody-l26-ab9475.html<br>anti-human CD4 (rabbit; ZSbio, Cat. no. ZA-0519)<br>http://www.zsbio.com/product/ZA-0519<br>anti-human CD8A (mouse; ZSbio, Cat. no. TA802079)<br>http://www.zsbio.com/product/TA802079<br>anti-human FAP-α (rabbit; Abcam, Cat. no. ab207178)<br>https://www.abcam.com/products/primary-antibodies/fibroblast-activation-protein-alpha-antibody-epr20021-ab207178.html<br>anti-human CASP3 (rabbit, CST, Cat. no. 9964)<br>https://www.cellsignal.com/products/primary-antibodies/cleaved-caspase-3-asp175-5a1e-rabbit-mab/9664<br>anti-human CD3 (rabbit; ZSbio, Cat no. ZA-0503)<br>http://www.zsbio.com/product/ZA-0503<br>anit-human CD68 (mouse; ZSbio, Cat no. TA802952),<br>http://www.zsbio.com/product/TA802952<br>anit-human CD56 (mouse; ZSbio, Cat no. ZM-0057),<br>http://www.zsbio.com/product/ZM-0057<br>anti-human PanCK (mouse; ZSbio, Cat no. ZM-0069)<br>http://www.zsbio.com/product/ZM-0069<br>anti-human CXCR5 (rabbit; Abcam, Cat. no. ab254415)<br>https://www.abcam.com/products/primary-antibodies/cxcr5-antibody-epr23463-30-ab254415.html<br>anti-human PD1 (mouse; ZSbio, Cat no. ZM-0381)<br>http://www.zsbio.com/product/ZM-0381<br>anti-human TIM3 (rabbit; CST, Cat. no. 45208)<br>https://www.cellsignal.com/products/primary-antibodies/tim-3-d5d5r-xp-rabbit-mab/45208<br>anti-human IgG(rabbit; ZSbio, Cat no. ZA-0448)<br>http://www.zsbio.com/product/ZA-0448<br>anti-human IgA(rabbit; ZSbio, Cat no. ZA-0446)<br>http://www.zsbio.com/product/ZA-0446<br>anti-human E-Cadherin (mouse; CST, Cat. no. CST14472)<br>https://www.cellsignal.com/products/primary-antibodies/e-cadherin-4a2-mouse-mab/14472

anti-human VCAM1(mouse; BioLegend, Cat. no. 305805)
https://www.biolegend.com/fr-lu/products/pe-anti-human-cd106-antibody-842?GroupID=BLG10116
anti-rabbit IgG (goat; CST, Cat. no. 7074)
https://www.cellsignal.com/products/secondary-antibodies/anti-rabbit-igg-hrp-linked-antibody/7074
anti-mouse IgG (horse; CST, Cat. no. 7076),
https://www.cellsignal.com/products/secondary-antibodies/anti-mouse-igg-hrp-linked-antibody/7076
anti-human GAPDH (mouse; ABclonal, Cat. no. AC002)
https://abclonal.com/catalog-antibodies/GAPDHMousemAb/AC002
anti-human CXCL13 (rabbit, NOVUS, Cat. no. NBP2-16041)
https://www.novusbio.com/products/cxcl13-blc-bca-1-antibody_nbp2-16041
anti-CD70 (rabbit, CST, Cat. no. 69209)
https://www.cellsignal.com/products/primary-antibodies/cd70-e3q1a-rabbit-mab/69209
anti-CD27 (rabbit, proteintech, Cat. no. 66308-1-lg).
https://www.ptglab.com/products/CD27-Antibody-66308-1-Ig.htm

Eukaryotic cell lines

Policy information about cell lines and Sex and Gender in Research

Cell line source(s): Human lung fibroblast cells (MRC-5) were obtained from Zhejiang Meisen Cell Technology Co., Ltd. . Human embryonic kidney 293T cells were obtained from Cell Bank of Type Culture Collection of the Chinese Academy of Sciences, Shanghai Institute of Cell Biology, Chinese Academy of Sciences. Human NPC cell lines (HK-1 and S26) were kindly gifted by Professor Chaonan Qian.

Authentication: Cell lines were authenticated by short tandem repeat (STR) fingerprinting.

Mycoplasma contamination: All cell lines were tested negative for mycoplasma contamination.

Commonly misidentified lines (See ICLAC register): None

Plants

Seed stocks: *Report on the source of all seed stocks or other plant material used. If applicable, state the seed stock centre and catalogue number. If plant specimens were collected from the field, describe the collection location, date and sampling procedures.*

Novel plant genotypes: *Describe the methods by which all novel plant genotypes were produced. This includes those generated by transgenic approaches, gene editing, chemical/radiation-based mutagenesis and hybridization. For transgenic lines, describe the transformation method, the number of independent lines analyzed and the generation upon which experiments were performed. For gene-edited lines, describe the editor used, the endogenous sequence targeted for editing, the targeting guide RNA sequence (if applicable) and how the editor was applied.*

Authentication: *Describe any authentication procedures for each seed stock used or novel genotype generated. Describe any experiments used to assess the effect of a mutation and, where applicable, how potential secondary effects (e.g. second site T-DNA insertions, mosiacism, off-target gene editing) were examined.*

Flow Cytometry

Plots

Confirm that:

- [x] The axis labels state the marker and fluorochrome used (e.g. CD4-FITC).
- [x] The axis scales are clearly visible. Include numbers along axes only for bottom left plot of group (a 'group' is an analysis of identical markers).
- [x] All plots are contour plots with outliers or pseudocolor plots.
- [x] A numerical value for number of cells or percentage (with statistics) is provided.

Methodology

Sample preparation: Tumour tissues were cut into small pieces, followed by collagenase digestion and filter by 40-μm cell-strainer. PBMCs were isolated using leukocyte separation solution. After lysing red blood cells and washing twice with DPBS, the dissociated cells from tumour and blood peripheral were resuspended in 0.04% BSA.

Instrument: BD FACSAria III, BD Biosciences, USA

Software: BD FACS Diva version 8

Cell population abundance: For cell sorting, an aliquot of cells was taken immediately after sorting and re-analyze at the same instrument. A

representative analysis is shown in Supplementary figure 12.

Gating strategy	Gating strategies used for sorting experiments are shown in Supplementary figure 12. Gate boundaries were set either based on control samples or followed density distribution on best practices.

☒ Tick this box to confirm that a figure exemplifying the gating strategy is provided in the Supplementary Information.
